# Supplementary material for: Acquisition of T6SS Effector TseL Contributes to the Emerging of Novel Epidemic Strains of Pseudomonas aeruginosa
Source: Microbiol Spectr. 2022 Dec 22;11(1):e03308-22. doi: 10.1128/spectrum.03308-22 (PMC9927574; doi:10.1128/spectrum.03308-22)
Supplement: Supplemental file 1 — Supplemental material. Download spectrum.03308-22-s0001.pdf, PDF file, 1.8 MB [file spectrum.03308-22-s0001.pdf]

**Acquisition of T6SS effector TseL contributes to the emerging of novel epidemic strains of *Pseudomonas aeruginosa***

Anmin Ren[1#], Minlu Jia[1#], Jihong Liu[3], Tian Zhou[1], Liwen Wu[2], Tao Dong[2], Zhao Cai[1], Jiuxin Qu[6], Yang Liu[3], Liang Yang[1,4,5*], Yingdan Zhang[1*]

[1]*School of Medicine, Southern University of Science and Technology, Shenzhen 518055, Guangdong, China*

[2]*School of Life Sciences, Southern University of Science and Technology, Shenzhen 518055, Guangdong, China*

[3]*Medical Research Center, Southern University of Science and Technology Hospital, Shenzhen 518055, Guangdong, China*

[4]*Shenzhen Third People's Hospital, The Second Affiliated Hospital of Southern University of Science and Technology, National Clinical Research Center for Infectious Disease, Shenzhen 518112, China*

[5]*Shenzhen Key Laboratory of Gene Regulation and Systems Biology, Southern University of Science and Technology, Shenzhen 518055, China*

[6]*The Second Affiliated Hospital of Southern University of Science and Technology, Shenzhen 518112, China*

[#] Authors contribute equally to this work. Author order was determined by drawing straws.

[*] E-mail to Liang Yang: yangl@sustech.edu.cn

[*] E-mail to Yingdan Zhang: zhangyd6@sustech.edu.cn

**Running title:** T6SS-effector TseL shapes epidemic strain of *P. aeruginosa*

**Key words:** *Pseudomonas aeruginosa*, T6SS effector, TseL, pathogenesis

**Supplementary method**

**In-frame deletion of target genes**

For gene in-frame deletion, the upstream and downstream DNA fragments of target gene were amplified with two pairs of primers F1/R1 and F2/R2, respectively (Table S2). The two PCR products purified by HiPure PCR pure mini kit (Magen) and ligated to Hind III and EcoR I double digested suicide vector PK18 by Gibson Assembly master mix (NEB) to yield PK18-target gene. After sequence confirmation, the suicide plasmid PK18-tanget gene was mobilized from E. coli DH5α (donor strain) to P. aeruginosa LYSZa7 (receptor strain) by conjugal mating with the help of pRK600 vector and selection for gentamycin-resistant first homologous recombinants. Colonies were then streaked on LB agar plates with 15% sucrose to select second homologous recombinants. And the target gene deletion mutant was identified by PCR.

**Table S1. Bacterial strains and plasmids used in this work**

| Strain/Plasmid | Characteristics/Application | Source/Reference |
|---|---|---|
| ***E. coli*** | | |
| TOP10 | Plasmid maintenance | Lab stock |
| DH5α | Plasmid maintenance | Lab stock |
| BL21(DE3) pLysS | Expression strain for toxic and non-toxic protein | Novagen |
| BL21(DE3) | Expression strain for toxic and non-toxic protein | Novagen |
| RK600 | Helper strain | Lab stock |
| ***P. aeruginosa strain*** | | |
| PAO1 | Wild type | Lab stock |
| PAK | Wild type | Lab stock |
| PA14 | Wild type | Lab stock |
| PDO300 | Wild type | Lab stock |
| LYSZa7 | Wild type | Lab stock |
| LYSZa7 *ΔtseL* | *tseL* deletion *mutant* | This study |
| LYSZa7 *ΔtseL::tseL* | Strain LYSZa7 *ΔtseL* carrying chromosomal integrated tetracycline resistant gene and *tseL* (MiniCTX-1-*tseL*) | This study |
| LYSZa7 *ΔtsiP1ΔtsiP2* | *tsiP1 and tsiP2* deletion mutant | This study |
| LYSZa7 *ΔtsiP1ΔtsiP2*::Tc | Strain LYSZa7 *ΔtsiP1ΔtsiP2* carrying chromosomal integrated tetracycline resistant gene (MiniCTX-1) | This study |
| LYSZa7 *ΔtsiP1ΔtsiP2*::*tsiP1* | Strain LYSZa7 *ΔtsiP1ΔtsiP2* carrying chromosomal integrated tetracycline resistant gene and *tsiP1* (MiniCTX-1-*tsiP1*) | This study |
| LYSZa7 *ΔtsiP1ΔtsiP2*::*tsiP2* | Strain LYSZa7 *ΔtsiP1ΔtsiP2* carrying chromosomal integrated tetracycline resistant gene and *tsiP2* (MiniCTX-1-*tsiP2*) | This study |
| LYSZa7*ΔtseLΔtsiP1ΔtsiP2* | *tseL*, *tsiP1* and *tsiP2* deletion mutant | This study |

| | | |
|---|---|---|
| LYSZa7*ΔtssM1tssM2tssB3tssM4* | T6SS defective mutant.<br>*tssM1*, *tssM2*, *tssB3* and *tssM4* deletion mutant, which is defective in T6SS function. | This study |
| ***K. pneumoniae* strain** | | |
| BAA1705 | Wild type | Lab stock |
| ***A. Baumanii strain*** | | |
| ATCC17978 | Wild type | Lab stock |
| **Plasmids** | | |
| pET28 a(+) | plasmid vector for IPTG-inducible expression of target genes | Novagen |
| pET28-tseL | pET28 a(+) expressing *tseL* | This study |
| pET22 b(+) | plasmid vector for IPTG-inducible expression of target genes, with directed periplasmic translocation | Novagen |
| pET22-*tseL* | pET22 b(+) expressing periplasm-targeted *tseL* | This study |
| pET22-*tseL* H466A | pET22 b(+) expressing periplasm-targeted *tseL*$^{H466A}$ mutant | This study |
| pET22-*tseL*-i1 | pET22 b(+) expressing genes tseL and *tsiP*1 | This study |
| pET22-*tseL*-i2 | pET22 b(+) expressing genes tseL and *tsiP*2 | This study |
| pET22-tseL-i3 | pET22 b(+) expressing genes *tseL, tsiP*2 and *tsiP*1 | This study |
| pBBR1MCS5 | Plasmid vector with broad host-range | Kovach et al., 1995 |
| pME6032 | IPTG-inducible expressing vector with broad host-range | Tian et al., 2009 |
| pME6032-*tseL* | pME6032 expressing *tseL* with C-terminal FLAG tag | This study |
| pBAD/Myc-His A | Expressing vector with C-terminal Myc-His tag | Invitrogen |
| pBAD-1 | pBAD/Myc-His A expressing *tsiP*1 | This study |
| pBAD-2 | pBAD/Myc-His A expressing *tsiP*2 | This study |
| pET28 a(+)-sumo | Expression vector for producing SUMO fusion protein cytoplasmically | This study |
| pET28 a-sumo-*tseL* | pET28 a(+)-sumo expressing SUMO-fused TseL | This study |
| pET28 a-sumo-*tseL*$^{H466A}$ | pET28 a(+)-sumo expressing SUMO-fused TseL$^{H466A}$ mutant | This study |
| mini-CTX1 | Expression vectors that can be integrated into the genome of *P.aeruginosa* | Tung T.et al.,1999 |

| | | |
|---|---|---|
| mini-CTX-*tseL* | mini-CTX1 expressing *tseL* gene | This study |
| mini-CTX-*tsiP*1 | mini-CTX1 expressing *tsiP*1 gene | This study |
| mini-CTX-*tsiP*2 | mini-CTX1 expressing *tsiP2* gene | This study |
| pEXG2.0 | For construction of *tssM* mutant. | Joseph D. M. et al., 2006 |
| pK18mobsacb | Suicide vector consisting of SacB gene which is sensitive to sucrose | Schäfer A.et al.,1994 |
| pK18-*tseL* | For generating knockout of *tseL* | This study |
| pK18-*tsiP*1-*tsiP2* | For generating knockout of *tseL*, *tsiP*2 and *tsiP*1 | This study |

**Table S2. Primers used in this work**

| Name | Sequence (5'-3') | Characteristics/Application |
|---|---|---|
| 22bt-f | TGGATATCGGAATTAATTCCGATCCATGATTTTGGGGCCGATGGAG | to amplify *tseL* onto pET22b(+) vector |
| 22bt-r | TCGAGTGCGGCCGCAAGCTTCTATCCAAGCGTATCGATGTCCAG | |
| 28at-f | GACAGCAAATGGGTCGCGGATCCATGATTTTGGGGCCGATGGA | to amplify *tseL* onto pET28a(+) vector |
| 28at-r | GGTGCTCGAGTGCGGCCGCAAGCTTCTATCCAAGCGTATCGATGT | |
| 22b-v-f | CGGCGTAGAGGATCGAGATC | pET22b (+) vector validation |
| 22b-v-r | TTATTGCTCAGCGGTGGCAG | |
| 28a-v-f | GGGGAATTGTGAGCGGATAA | pET28a (+) vector validation |
| 28a-v-r | CCCCAAGGGGTTATGCTAGT | |
| 22bti1-f | ATGAACCGCACCCTTAAAAGCATG | to amplify *tsiP1* |
| 22bti1-r | TCATGGGGTCACCACCTTGTTTTCCGTA | |
| 22bti2-f | ATGAACCGCACCCTTAAAAACATGACC | to amplify *tsiP2* |
| 22bti2-r | TTATTCGTCCACGGGTTGAAAGTTGCT | |
| H466A-f | CAGGCCTCTGGTAGCTCACCGCATGGTCA | for TseL site-directed mutations |
| H466R-r | TGACCATGCGGTGAGCTACCAGAGGCCTG | |
| flagt-f | AATTTCACACAGGAAACAGAATTCATGATTTTGGGGCCGATGGA | to amplify *tseL* onto pME6032 with flag label |
| flagt-r | CTAGTCCGAGGCCTCGAGCTTGTCATCGTCGTCCTTGTAATCTCCAAGCGTATCGATGTCCAGATC | |
| pME6032-f | CAGGCCGAATCAATCGGCAC | pME6032 vector validation |
| pME6032-r | TGGTGCCGAGGCGGAAAGTA | |
| pBADi1-f | ATTAACCATGGATCCGAGCTCATGAACCGCACCCTTAAAAGCATG | to amplify *tsiP1* onto pBAD/Myc-His A vector |
| pBADi1-r | TTTTTGTTCGGGCCCAAGCTTTGGGGTCACCACCTTGTTTTCC | |
| pBADi2-f | ATTAACCATGGATCCGAGCTCATGAACCGCACCCTTAAAAACATGACC | to amplify *tsiP2* onto pBAD/Myc-His A vector |
| pBADi2-r | TTTTTGTTCGGGCCCAAGCTTTGGGGTCACCGCCTTGTTTTC | |

| | | |
|---|---|---|
| pBAD-v-f<br>pBAD-v-r | TCCATACCCGTTTTTTGGGCTAAC<br>TGAGATGAGTTTTTGTTCGGGCC | pBAD/Myc-His A vector validation |
| sumot-f<br>sumot-r | GAACAGATTGGTGGATCCATGATTTTGGGGCCGATGGAGG<br>GAGTGCGGCCGCAAGCTTCTATCCAAGCGTATCGATGTCCAGATCG | to amplify *tseL* onto pET28 a(+)-sumo vector |
| sumo-v-f<br>sumo-v-r | CCCCTGAAGATTTGGACATGGA<br>TTCCTTTCGGGCTTTGTTAGCAG | pET28 a(+)-sumo vector validation |
| minit-f<br>minit-r | TCGAGGTCGACGGTATCGATAAGCTTATGATTTTGGGGCCGATGGAGGAG<br>CGGCCGCTCTAGAACTAGTGGATCCCTATCCAAGCGTATCGATGTCCAGATCGAA | to amplify *tseL* onto mini-CTX1 vector |
| minii1-f<br>minii1-r | TCGAGGTCGACGGTATCGATAAGCTTATGAACCGCACCCTTAAAAGCATG<br>CGGCCGCTCTAGAACTAGTGGATCCTCATGGGGTCACCACCTTGTTTT | to amplify *tsiP1* onto mini-CTX1 vector |
| minii2-f<br>minii2-r | TCGAGGTCGACGGTATCGATAAGCTTATGAACCGCACCCTTAAAAACATGACC<br>CGGCCGCTCTAGAACTAGTGGATCCTCATGGGGTCACCGCCTTGTT | to amplify *tsiP2* onto mini-CTX1 vector |
| mini-v-f<br>mini-v-r | CCCTCGAGGTCGACGGTATCG<br>GGTGGCGGCCGCTCTAGAACT | mini-CTX1 vector validation |
| pK18-f<br>pK18-r | AGCTCGGTACCCGGGGATCC<br>CGACGGCCAGTGCCAAGCTT | pK18 homologous arm |
| tseLf1<br>tseLr1 | ATGTATATGGTTGGTATGGCAAGGATTGGG<br>TCATGGGGTCACCACCTTGTTTTCC | For *tseL* deletion |
| tseLf2<br>tseLr2 | AGCGGAGTCAAACAAACCCTATCGAGG<br>ACGCGGCATCTGCATCGAAGA | For *tseL* deletion |
| tsif1<br>tsir1 | ATCGCTTTCACGACAGTCGCGTG<br>TCATACGGGGGCGTCTCCTTGC | For *tsiP* deletion |
| tsif2<br>tsir2 | AGCGGAGTCAAACAAACCCTATCGAGG<br>ACGCGGCATCTGCATCGAAGA | For *tsiP* deletion |
| tssM1-f<br>tssM1-r | CCTGGGGCGGCGAGAAGGTCTTCCAGTTGCTCGAACACTGC<br>GCTCCGGGAGGAAGCCTTTCATTGCGC | For *tssM1* deletion |

| | | |
|---|---|---|
| tssM1-v-f | AAAGGCTTCCTCCCGGAGCGCCTTTGAACA | For *tssM1* deleted mutant verification |
| tssM1-v-r | CTGCAGGTCGACTCTGAGATCTACGATCAGGCTGCTGACGAA | |
| tssM2-f | TACCGAATTCGAGCTCGAGCGGAACTGAACATGATCAAGGAAAGGG | For *tssM2* deletion |
| tssM2-r | GCTGCACCGGGAAGAAGTTTTTCATTTCGCGTCCC | |
| tssM2-v-f | AAACTTCTTCCCGGTGCAGCTCTGATGCAA | For *tssM2* deleted mutant verification |
| tssM2-v-r | CTGCAGGTCGACTCTGAGATCTGACGACAGCGGTTCGTTCATT | |
| tssB3-f | TACCGAATTCGAGCTCGAGCTCCCTGCAACTGGAAATGCTG | For *tssB3* deletion |
| tssB3-r | GGAAGAGGGTCACAGCTTGTGCTGCGTACTCTC | |
| tssB3-v-f | CAGCACAAGCTGTGACCCTCTTCCCGGAGAAG | For *tssB3* deleted mutant verification |
| tssB3-v-r | CTGCAGGTCGACTCTGAGATCTCGTCCTCGACATAGTTCATGCCTT | |
| tssM4-f | TACCGAATTCGAGCTCGAGCAGCATGCCGAATCCAATCGCG | For *tssM4* deletion |
| tssM4-r | CAGCCTCGCGCCAGATTTTCCACATGGCAATGC | |
| tssM4-v-f | GTGGAAAATCTGGCGCGAGGCTGGATAAGATGC | For *tssM4* deleted mutant verification |
| tssM4-v-r | CTGCAGGTCGACTCTGAGATCTATCATCTCGATGCCGTCGTGC | |

**Table S3 Dysregulated genes in E. coli BL21 (DE3) by the expression of TseL.**

| geng_id | $\log_2$FoldChange | p-value | p-adjusted | Dysregulation | product |
|---|---|---|---|---|---|
| abgA | -1.46 | 1.42E-36 | 3.02E-34 | down | p-aminobenzoyl-glutamate hydrolase subunit A |
| abgB | -1.55 | 4.37E-36 | 8.79E-34 | down | p-aminobenzoyl-glutamate hydrolase subunit B |
| abgT | -1.27 | 7.60E-19 | 3.73E-17 | down | p-aminobenzoyl glutamate:H+ symporter |
| aceA | -1.16 | 1.40E-05 | 1.06E-04 | down | isocitrate lyase |
| aceK | -1.82 | 9.02E-10 | 1.51E-08 | down | isocitrate dehydrogenase kinase/phosphatase |
| acs | -1.01 | 2.44E-10 | 4.53E-09 | down | acetyl-CoA synthetase (AMP-forming) |
| actP | -1.38 | 2.02E-11 | 4.53E-10 | down | acetate/glycolate:cation symporter |
| agaV | -1.14 | 7.28E-05 | 4.75E-04 | down | N-acetyl-D-galactosamine specific PTS enzyme IIB component |
| aldA | -1.09 | 1.65E-20 | 9.79E-19 | down | aldehyde dehydrogenase A |
| allB | -1.18 | 2.59E-06 | 2.26E-05 | down | allantoinase |
| alsA | -1.02 | 1.47E-07 | 1.62E-06 | down | D-allose ABC transporter ATP binding subunit |
| alsC | -1.76 | 4.43E-23 | 3.36E-21 | down | D-allose ABC transporter membrane subunit |
| alsE | -2.24 | 7.57E-43 | 2.18E-40 | down | D-allulose-6-phosphate 3-epimerase |
| alsK | -1.92 | 1.82E-26 | 1.83E-24 | down | D-allose kinase |
| ansB | -1.03 | 1.89E-07 | 2.04E-06 | down | L-asparaginase 2 |
| araA | -1.53 | 7.76E-17 | 2.97E-15 | down | L-arabinose isomerase |
| araB | -1.11 | 4.98E-08 | 5.94E-07 | down | ribulokinase |
| araC | -1.73 | 1.64E-21 | 1.12E-19 | down | DNA-binding transcriptional dual regulator AraC |
| araD_1 | -1.25 | 4.68E-06 | 3.86E-05 | down | |
| araF | -1.52 | 2.29E-24 | 2.05E-22 | down | arabinose ABC transporter periplasmic binding protein |
| argO | -1.14 | 7.65E-08 | 8.87E-07 | down | L-arginine exporter |
| arnE | -1.17 | 1.17E-13 | 3.34E-12 | down | undecaprenyl-phosphate-alpha-L-Ara4N flippase - ArnE subunit |
| astB | -1.77 | 1.93E-11 | 4.36E-10 | down | N-succinylarginine dihydrolase |
| astD | -1.31 | 2.57E-08 | 3.22E-07 | down | aldehyde dehydrogenase |
| astE | -2.51 | 6.75E-17 | 2.66E-15 | down | succinylglutamate desuccinylase |

| | | | | | |
|---|---|---|---|---|---|
| atpD | -1.24 | 9.32E-13 | 2.39E-11 | down | ATP synthase F1 complex subunit beta |
| betA | -1.80 | 1.22E-26 | 1.26E-24 | down | choline dehydrogenase |
| betB | -1.64 | 3.65E-24 | 3.13E-22 | down | betaine aldehyde dehydrogenase |
| betI | -1.46 | 2.78E-18 | 1.27E-16 | down | DNA-binding transcriptional repressor BetI |
| betT_1 | -1.08 | 9.32E-10 | 1.54E-08 | down | |
| bglJ | -1.72 | 3.49E-04 | 1.87E-03 | down | DNA-binding transcriptional regulator BglJ |
| bioA | -1.85 | 7.13E-20 | 4.04E-18 | down | adenosylmethionine-8-amino-7-oxononanoate aminotransferase |
| bioB | -2.20 | 3.20E-23 | 2.48E-21 | down | biotin synthase |
| bioC | -1.99 | 5.01E-21 | 3.20E-19 | down | malonyl-acyl carrier protein methyltransferase |
| bioD_1 | -1.63 | 5.96E-20 | 3.42E-18 | down | |
| bioF | -2.29 | 2.14E-21 | 1.41E-19 | down | 8-amino-7-oxononanoate synthase |
| bioP | -1.10 | 5.73E-09 | 8.06E-08 | down | biotin transporter |
| btsT | -2.27 | 3.52E-29 | 4.73E-27 | down | pyruvate:H+ symporter |
| cadA | -4.06 | 4.80E-77 | 2.76E-74 | down | lysine decarboxylase 1 |
| cadB | -3.58 | 5.85E-53 | 2.36E-50 | down | lysine:cadaverine antiporter |
| clpB | -1.61 | 4.29E-16 | 1.56E-14 | down | chaperone protein ClpB |
| cysA | -1.14 | 1.36E-05 | 1.04E-04 | down | sulfate/thiosulfate ABC transporter ATP binding subunit |
| cysC | -1.45 | 1.35E-07 | 1.49E-06 | down | adenylyl-sulfate kinase |
| cysH | -1.09 | 1.11E-06 | 1.04E-05 | down | phosphoadenosine phosphosulfate reductase |
| dctA | -1.05 | 5.86E-16 | 2.09E-14 | down | C4 dicarboxylate/orotate:H(+) symporter |
| dgoA | -1.00 | 6.67E-06 | 5.32E-05 | down | 2-dehydro-3-deoxy-6-phosphogalactonate aldolase |
| dgoD | -1.11 | 4.85E-07 | 4.93E-06 | down | D-galactonate dehydratase |
| dgoK | -1.02 | 7.50E-11 | 1.52E-09 | down | 2-dehydro-3-deoxygalactonokinase |
| dnaK | -1.41 | 8.74E-17 | 3.32E-15 | down | chaperone protein DnaK |
| dppF | -1.03 | 3.26E-04 | 1.76E-03 | down | dipeptide ABC transporter ATP binding subunit DppF |
| dsdA | -3.89 | 8.16E-112 | 1.10E-108 | down | D-serine ammonia-lyase |
| dsdC | -1.60 | 4.62E-17 | 1.86E-15 | down | DNA-binding transcriptional dual regulator DsdC |

| | | | | | |
|---|---|---|---|---|---|
| dsdX | -5.45 | 5.22E-126 | 2.10E-122 | down | D-serine transporter |
| dtpC | -1.13 | 1.20E-08 | 1.61E-07 | down | dipeptide/tripeptide:H+ symporter DtpC |
| ECD_RS00080 | -1.72 | 4.25E-02 | 1.05E-01 | down | |
| ECD_RS00105 | -1.82 | 2.80E-02 | 7.58E-02 | down | |
| ECD_RS01125 | -1.11 | 6.44E-04 | 3.24E-03 | down | |
| ECD_RS01210 | -5.04 | 3.31E-02 | NA | down | |
| ECD_RS01335 | -1.58 | 1.03E-04 | 6.43E-04 | down | |
| ECD_RS02195 | -1.25 | 7.70E-06 | 6.04E-05 | down | |
| ECD_RS02625 | -1.91 | 6.98E-14 | 2.04E-12 | down | |
| ECD_RS02630 | -1.37 | 5.37E-17 | 2.14E-15 | down | |
| ECD_RS02775 | -2.43 | 2.63E-02 | NA | down | |
| ECD_RS03105 | -2.05 | 3.94E-02 | 9.87E-02 | down | |
| ECD_RS04310 | -1.74 | 4.67E-08 | 5.62E-07 | down | |
| ECD_RS04545 | -1.03 | 2.93E-09 | 4.38E-08 | down | |
| ECD_RS05255 | -2.26 | 2.38E-02 | 6.68E-02 | down | |
| ECD_RS07140 | -1.08 | 2.67E-07 | 2.83E-06 | down | |
| ECD_RS07525 | -1.11 | 3.96E-05 | 2.74E-04 | down | |
| ECD_RS07750 | -1.99 | 1.75E-29 | 2.52E-27 | down | |
| ECD_RS08100 | -1.34 | 1.48E-02 | 4.48E-02 | down | |
| ECD_RS09130 | -1.23 | 2.80E-05 | 2.00E-04 | down | |
| ECD_RS09140 | -2.04 | 1.09E-15 | 3.73E-14 | down | |
| ECD_RS09145 | -1.88 | 3.27E-12 | 7.92E-11 | down | |
| ECD_RS09150 | -1.77 | 6.95E-18 | 3.11E-16 | down | |
| ECD_RS09180 | -1.48 | 1.26E-17 | 5.38E-16 | down | |
| ECD_RS10200 | -1.42 | 2.28E-03 | 9.36E-03 | down | |
| ECD_RS10480 | -1.43 | 9.05E-09 | 1.23E-07 | down | |
| ECD_RS10485 | -1.08 | 1.61E-13 | 4.57E-12 | down | |

| | | | | |
|---|---|---|---|---|
| ECD_RS10495 | -1.21 | 4.70E-14 | 1.41E-12 | down |
| ECD_RS10620 | -1.04 | 3.30E-02 | 8.61E-02 | down |
| ECD_RS12055 | -1.05 | 7.37E-03 | 2.55E-02 | down |
| ECD_RS12635 | -1.14 | 2.30E-09 | 3.50E-08 | down |
| ECD_RS12670 | -1.08 | 7.99E-07 | 7.75E-06 | down |
| ECD_RS12675 | -1.27 | 2.28E-07 | 2.44E-06 | down |
| ECD_RS12680 | -1.33 | 1.40E-09 | 2.24E-08 | down |
| ECD_RS12685 | -1.78 | 3.93E-11 | 8.40E-10 | down |
| ECD_RS13565 | -1.06 | 1.56E-04 | 9.30E-04 | down |
| ECD_RS13800 | -6.89 | 1.99E-06 | NA | down |
| ECD_RS13805 | -2.54 | 1.07E-28 | 1.31E-26 | down |
| ECD_RS13810 | -1.02 | 2.15E-08 | 2.76E-07 | down |
| ECD_RS13820 | -1.16 | 5.36E-16 | 1.93E-14 | down |
| ECD_RS14665 | -1.10 | 3.74E-09 | 5.43E-08 | down |
| ECD_RS14675 | -1.22 | 2.99E-08 | 3.70E-07 | down |
| ECD_RS14680 | -1.68 | 3.02E-10 | 5.42E-09 | down |
| ECD_RS14685 | -1.95 | 3.11E-09 | 4.62E-08 | down |
| ECD_RS14690 | -3.11 | 3.83E-29 | 4.97E-27 | down |
| ECD_RS14695 | -2.75 | 2.91E-42 | 7.80E-40 | down |
| ECD_RS14700 | -2.15 | 1.70E-19 | 9.11E-18 | down |
| ECD_RS14705 | -1.31 | 3.27E-14 | 1.00E-12 | down |
| ECD_RS14825 | -1.28 | 4.88E-13 | 1.27E-11 | down |
| ECD_RS15235 | -1.13 | 9.24E-22 | 6.64E-20 | down |
| ECD_RS15495 | -1.06 | 1.89E-06 | 1.69E-05 | down |
| ECD_RS15520 | -1.15 | 3.67E-10 | 6.46E-09 | down |
| ECD_RS16050 | -1.09 | 1.97E-08 | 2.54E-07 | down |
| ECD_RS17400 | -1.24 | 9.61E-09 | 1.30E-07 | down |

| | | | | | |
|---|---|---|---|---|---|
| ECD_RS17410 | -1.31 | 4.66E-10 | 8.09E-09 | down | |
| ECD_RS17520 | -2.68 | 1.42E-05 | 1.08E-04 | down | |
| ECD_RS17850 | -3.33 | 1.20E-02 | NA | down | |
| ECD_RS18375 | -1.68 | 4.34E-26 | 4.15E-24 | down | |
| ECD_RS19720 | -1.14 | 1.15E-04 | 7.10E-04 | down | |
| ECD_RS20105 | -1.28 | 1.82E-05 | 1.34E-04 | down | |
| ECD_RS20450 | -2.93 | 6.87E-17 | 2.67E-15 | down | |
| ECD_RS20735 | -1.30 | 7.04E-10 | 1.19E-08 | down | |
| ECD_RS21130 | -1.26 | 5.53E-09 | 7.81E-08 | down | |
| ECD_RS21460 | -1.05 | 9.56E-03 | 3.12E-02 | down | |
| ECD_RS21815 | -1.30 | 4.89E-10 | 8.45E-09 | down | |
| ECD_RS21820 | -1.37 | 1.71E-13 | 4.81E-12 | down | |
| ECD_RS22010 | -1.78 | 2.25E-09 | 3.46E-08 | down | |
| ECD_RS22205 | -1.02 | 2.13E-02 | 6.08E-02 | down | |
| ECD_RS22265 | -1.62 | 7.16E-16 | 2.53E-14 | down | |
| ECD_RS22400 | -1.48 | 1.47E-04 | 8.80E-04 | down | |
| ECD_RS24960 | -5.94 | 4.30E-04 | NA | down | |
| ECD_RS25270 | -1.09 | 3.70E-04 | 1.97E-03 | down | |
| ECD_RS26055 | -1.27 | 3.95E-02 | 9.87E-02 | down | |
| entA | -1.10 | 1.21E-07 | 1.36E-06 | down | 2,3-dihydro-2,3-dihydroxybenzoate dehydrogenase |
| entB | -1.65 | 1.62E-14 | 5.09E-13 | down | enterobactin synthase component B |
| entC | -1.88 | 4.67E-13 | 1.23E-11 | down | isochorismate synthase EntC |
| entD | -2.65 | 2.31E-13 | 6.42E-12 | down | phosphopantetheinyl transferase EntD |
| entE | -1.68 | 1.41E-14 | 4.50E-13 | down | 2,3-dihydroxybenzoate-AMP ligase |
| entF | -2.11 | 3.87E-21 | 2.51E-19 | down | holo [EntF peptidyl-carrier protein] |
| entH | -1.08 | 1.06E-03 | 4.86E-03 | down | proofreading thioesterase in enterobactin biosynthesis |
| entS | -1.11 | 1.14E-06 | 1.07E-05 | down | enterobactin exporter EntS |

| | | | | | |
|---|---|---|---|---|---|
| espX5 | -1.89 | 5.22E-06 | 4.24E-05 | down | |
| fadA | -1.58 | 2.39E-30 | 3.56E-28 | down | 3-ketoacyl-CoA thiolase |
| fadB | -1.03 | 1.13E-10 | 2.22E-09 | down | multifunctional enoyl-CoA hydratase, 3-hydroxyacyl-CoA epimerase, Delta3-cis-Delta2-trans-enoyl-CoA isomerase, L-3-hydroxyacyl-CoA dehydrogenase |
| fadE | -1.47 | 5.27E-33 | 8.48E-31 | down | acyl-CoA dehydrogenase |
| fecC | -1.45 | 1.30E-07 | 1.45E-06 | down | ferric citrate ABC transporter membrane subunit FecC |
| fecD | -1.71 | 5.49E-10 | 9.33E-09 | down | ferric citrate ABC transporter membrane subunit FecD |
| fecE | -1.78 | 2.53E-11 | 5.56E-10 | down | ferric citrate ABC transporter ATP binding subunit |
| fecR | -1.09 | 4.96E-06 | 4.05E-05 | down | ferric citrate regulator FecR |
| fepA | -2.53 | 1.11E-21 | 7.84E-20 | down | ferric enterobactin outer membrane transporter |
| fepB | -1.43 | 1.40E-06 | 1.29E-05 | down | ferric enterobactin ABC transporter periplasmic binding protein |
| fepC | -1.36 | 9.59E-07 | 9.08E-06 | down | ferric enterobactin ABC transporter ATP binding subunit |
| fepD | -1.32 | 3.43E-05 | 2.41E-04 | down | ferric enterobactin ABC transporter membrane subunit FebD |
| fepG | -1.27 | 3.47E-05 | 2.43E-04 | down | ferric enterobactin ABC transporter membrane subunit FepG |
| fes | -2.18 | 2.74E-23 | 2.21E-21 | down | ferric enterobactin esterase |
| fhuA | -1.74 | 1.20E-10 | 2.33E-09 | down | ferrichrome outer membrane transporter/phage receptor |
| fhuB | -1.34 | 1.05E-07 | 1.20E-06 | down | iron(III) hydroxamate ABC transporter membrane subunit |
| fhuC | -1.46 | 6.26E-08 | 7.32E-07 | down | iron(III) hydroxamate ABC transporter ATP binding subunit |
| fhuD | -1.75 | 7.64E-11 | 1.54E-09 | down | iron(III) hydroxamate ABC transporter periplasmic binding protein |
| fhuE | -1.19 | 3.21E-09 | 4.75E-08 | down | ferric coprogen/ferric rhodotorulic acid outer membrane transporter |
| fhuF | -1.83 | 2.23E-11 | 4.98E-10 | down | ferric-siderophore reductase FhuF |
| frlA | -1.58 | 4.14E-08 | 5.06E-07 | down | fructoselysine/psicoselysine transporter |
| frlB | -1.14 | 4.56E-11 | 9.66E-10 | down | fructoselysine 6-phosphate deglycase |
| fucI | -1.12 | 3.80E-16 | 1.40E-14 | down | L-fucose isomerase |
| fucK | -1.16 | 3.16E-23 | 2.48E-21 | down | L-fuculokinase |
| gabP | -1.14 | 1.06E-10 | 2.11E-09 | down | 4-aminobutanoate:H+ symporter |
| gadE | -1.02 | 2.66E-04 | 1.48E-03 | down | DNA-binding transcriptional activator GadE |

| | | | | | |
|---|---|---|---|---|---|
| gap | -1.25 | 4.31E-16 | 1.56E-14 | down | |
| garD | -1.14 | 1.08E-18 | 5.20E-17 | down | GarD |
| garK | -1.89 | 2.00E-19 | 1.06E-17 | down | glycerate 2-kinase 1 |
| garL | -2.05 | 4.65E-52 | 1.56E-49 | down | alpha-dehydro-beta-deoxy-D-glucarate aldolase |
| garP | -1.87 | 2.27E-29 | 3.16E-27 | down | galactarate/D-glucarate transporter GarP |
| garR | -2.13 | 1.68E-34 | 3.07E-32 | down | tartronate semialdehyde reductase |
| gatB | -1.26 | 5.73E-11 | 1.19E-09 | down | galactitol-specific PTS enzyme IIB component |
| gatZ | -1.03 | 9.32E-11 | 1.87E-09 | down | putative tagatose-1,6-bisphosphate aldolase 2 chaperone |
| ghoS_2 | -3.16 | 1.34E-03 | NA | down | |
| glcC | -1.02 | 3.56E-19 | 1.81E-17 | down | DNA-binding transcriptional dual regulator GlcC |
| glcD | -2.02 | 8.43E-18 | 3.69E-16 | down | glycolate dehydrogenase%2C putative FAD-linked subunit |
| glcE | -1.58 | 1.21E-10 | 2.34E-09 | down | glycolate dehydrogenase, putative FAD-binding subunit |
| glgS | -1.39 | 1.88E-06 | 1.69E-05 | down | surface composition regulator |
| glpA | -1.05 | 2.32E-08 | 2.95E-07 | down | anaerobic glycerol-3-phosphate dehydrogenase subunit A |
| glsA | -1.10 | 1.28E-07 | 1.43E-06 | down | glutaminase 1 |
| gltA | -1.21 | 7.97E-27 | 8.67E-25 | down | citrate synthase |
| glxR | -1.37 | 2.40E-03 | 9.76E-03 | down | tartronate semialdehyde reductase 2 |
| gntK | -1.78 | 2.53E-17 | 1.05E-15 | down | D-gluconate kinase%2C thermostable |
| gntT | -1.18 | 1.56E-09 | 2.47E-08 | down | high-affinity gluconate transporter |
| groL | -2.34 | 2.44E-31 | 3.77E-29 | down | chaperonin GroEL |
| gspI_1 | -1.33 | 3.38E-02 | 8.77E-02 | down | |
| gudP | -1.38 | 5.26E-12 | 1.27E-10 | down | galactarate/D-glucarate transporter GudP |
| hcxA | -1.76 | 4.56E-23 | 3.39E-21 | down | hydroxycarboxylate dehydrogenase A |
| hdhA | -1.45 | 6.41E-14 | 1.88E-12 | down | 7-alpha-hydroxysteroid dehydrogenase |
| hmpA | -1.39 | 4.75E-15 | 1.55E-13 | down | |
| hpaA | -1.55 | 4.67E-10 | 8.09E-09 | down | |
| hpaB | -1.12 | 9.06E-10 | 1.51E-08 | down | |

| | | | | | |
|---|---|---|---|---|---|
| hpaI | -1.26 | 5.87E-12 | 1.38E-10 | down | |
| hpaX | -1.69 | 1.70E-15 | 5.76E-14 | down | |
| hpf | -1.07 | 2.99E-11 | 6.50E-10 | down | ribosome hibernation-promoting factor |
| htpG | -1.83 | 3.04E-27 | 3.49E-25 | down | chaperone protein HtpG |
| hyi | -1.27 | 2.72E-02 | 7.41E-02 | down | hydroxypyruvate isomerase |
| idnD | -1.43 | 2.71E-24 | 2.37E-22 | down | L-idonate 5-dehydrogenase |
| idnO | -1.70 | 2.84E-25 | 2.66E-23 | down | 5-keto-D-gluconate 5-reductase |
| katE | -1.11 | 2.59E-10 | 4.76E-09 | down | catalase HPII |
| kdsB_2 | -1.11 | 5.98E-12 | 1.40E-10 | down | |
| lacI_1 | -1.12 | 1.41E-06 | 1.30E-05 | down | |
| mdtI | -1.00 | 3.23E-02 | 8.48E-02 | down | multidrug/spermidine efflux pump membrane subunit MdtI |
| melR | -1.07 | 3.76E-07 | 3.87E-06 | down | DNA-binding transcriptional dual regulator MelR |
| metA | -2.12 | 1.01E-23 | 8.49E-22 | down | homoserine O-succinyltransferase |
| metB | -2.41 | 4.89E-34 | 8.19E-32 | down | O-succinylhomoserine(thiol)-lyase / O-succinylhomoserine lyase |
| metE | -1.46 | 1.86E-12 | 4.64E-11 | down | cobalamin-independent homocysteine transmethylase |
| metF | -1.69 | 6.91E-17 | 2.67E-15 | down | 5,1-methylenetetrahydrofolate reductase |
| metI | -1.11 | 1.10E-09 | 1.80E-08 | down | L-methionine/D-methionine ABC transporter membrane subunit |
| metL | -1.17 | 8.20E-12 | 1.88E-10 | down | fused aspartate kinase/homoserine dehydrogenase 2 |
| metN | -1.41 | 2.84E-12 | 6.92E-11 | down | L-methionine/D-methionine ABC transporter ATP binding subunit |
| metR | -1.71 | 1.81E-21 | 1.22E-19 | down | DNA-binding transcriptional dual regulator MetR |
| nanC | -3.18 | 4.90E-27 | 5.48E-25 | down | N-acetylneuraminate outer membrane channel |
| nanM | -2.21 | 1.20E-24 | 1.10E-22 | down | N-acetylneuraminate mutarotase |
| nanT | -1.75 | 9.14E-20 | 5.04E-18 | down | N-acetylneuraminate:H+ symporter |
| napB | -1.48 | 1.57E-05 | 1.18E-04 | down | periplasmic nitrate reductase cytochrome c55 protein |
| napG | -1.61 | 1.72E-11 | 3.91E-10 | down | ferredoxin-type protein NapG |
| napH | -1.23 | 8.57E-07 | 8.23E-06 | down | ferredoxin-type protein NapH |
| odhB | -1.53 | 1.93E-15 | 6.36E-14 | down | |

| | | | | | |
|---|---|---|---|---|---|
| ompG | -1.78 | 1.00E-02 | 3.25E-02 | down | outer membrane porin G |
| ompN | -1.68 | 3.13E-03 | 1.22E-02 | down | outer membrane porin N |
| ompW | -1.83 | 1.14E-20 | 6.92E-19 | down | outer membrane protein W |
| osmB | -1.10 | 5.80E-04 | 2.94E-03 | down | osmotically-inducible lipoprotein OsmB |
| osmY | -1.42 | 2.45E-12 | 6.01E-11 | down | periplasmic chaperone OsmY |
| ptsP_3 | -1.26 | 2.08E-12 | 5.16E-11 | down | |
| rayT | -1.09 | 6.50E-05 | 4.29E-04 | down | REP-associated tyrosine transposase |
| rbbA | -1.71 | 1.03E-15 | 3.58E-14 | down | ribosome-associated ATPase |
| rfaY | -1.08 | 1.59E-03 | 6.83E-03 | down | |
| rhaB | -1.16 | 3.40E-04 | 1.83E-03 | down | rhamnulokinase |
| rhmD | -1.03 | 1.33E-10 | 2.57E-09 | down | L-rhamnonate dehydratase |
| rpnB | -1.10 | 3.99E-03 | 1.51E-02 | down | recombination-promoting nuclease RpnB |
| rutA | -1.21 | 2.12E-03 | 8.78E-03 | down | pyrimidine monooxygenase RutA |
| rutB | -1.25 | 1.24E-03 | 5.48E-03 | down | peroxyureidoacrylate/ureidoacrylate amidohydrolase |
| sdhA | -1.14 | 4.26E-17 | 1.73E-15 | down | succinate:quinone oxidoreductase%2C FAD binding protein |
| sdhB | -1.21 | 2.43E-19 | 1.27E-17 | down | succinate:quinone oxidoreductase, iron-sulfur cluster binding protein |
| sdhC | -1.43 | 7.31E-19 | 3.63E-17 | down | succinate:quinone oxidoreductase%2C membrane protein SdhC |
| sdhD | -1.25 | 1.25E-19 | 6.79E-18 | down | succinate:quinone oxidoreductase, membrane protein SdhD |
| slp | -1.90 | 6.38E-14 | 1.88E-12 | down | starvation lipoprotein |
| soxS | -1.05 | 2.35E-13 | 6.49E-12 | down | DNA-binding transcriptional dual regulator SoxS |
| spy | -1.14 | 1.17E-03 | 5.25E-03 | down | ATP-independent periplasmic chaperone |
| ssuA | -1.57 | 5.10E-08 | 6.07E-07 | down | aliphatic sulfonate ABC transporter periplasmic binding protein |
| ssuC | -1.35 | 3.79E-04 | 2.01E-03 | down | aliphatic sulfonate ABC transporter membrane subunit |
| ssuD | -1.45 | 1.25E-08 | 1.67E-07 | down | FMNH2-dependent alkanesulfonate monooxygenase |
| ssuE | -1.24 | 2.16E-04 | 1.26E-03 | down | NADPH-dependent FMN reductase |
| sucA | -1.23 | 1.95E-14 | 6.09E-13 | down | subunit of E1() component of 2-oxoglutarate dehydrogenase |
| sucC | -1.60 | 3.50E-13 | 9.40E-12 | down | succinyl-CoA synthetase subunit beta |

| | | | | | |
|---|---|---|---|---|---|
| sucD | -1.55 | 2.83E-13 | 7.64E-12 | down | succinyl-CoA synthetase subunit alpha |
| tauC | -1.15 | 2.19E-04 | 1.27E-03 | down | taurine ABC transporter membrane subunit |
| tauD | -1.46 | 1.17E-09 | 1.90E-08 | down | taurine dioxygenase |
| tnaA | -1.67 | 7.63E-41 | 1.92E-38 | down | tryptophanase |
| tnaB | -1.24 | 1.17E-09 | 1.90E-08 | down | tryptophan:H+ symporter TnaB |
| ugpB | -1.02 | 2.70E-19 | 1.39E-17 | down | sn-glycerol 3-phosphate ABC transporter periplasmic binding protein |
| ugpC | -1.08 | 1.90E-17 | 7.96E-16 | down | sn-glycerol 3-phosphate ABC transporter ATP binding subunit |
| uidA | -1.23 | 1.60E-17 | 6.77E-16 | down | beta-glucuronidase |
| uidB | -1.01 | 2.00E-09 | 3.12E-08 | down | glucuronide:H+ symporter |
| ulaA | -1.06 | 2.90E-09 | 4.36E-08 | down | L-ascorbate specific PTS enzyme IIC component |
| uxaA | -1.06 | 1.55E-10 | 2.95E-09 | down | D-altronate dehydratase |
| uxaC | -1.60 | 2.90E-18 | 1.31E-16 | down | D-glucoronate/D-galacturonate isomerase |
| ybdL | -1.82 | 1.53E-16 | 5.75E-15 | down | methionine transaminase |
| ybdZ | -2.46 | 2.34E-08 | 2.96E-07 | down | enterobactin biosynthesis protein YbdZ |
| ychH | -1.22 | 7.36E-11 | 1.50E-09 | down | stress-induced protein |
| ydjG | -1.94 | 5.82E-22 | 4.26E-20 | down | NADH-dependent methylglyoxal reductase |
| ygjG | -1.07 | 2.41E-12 | 5.95E-11 | down | |
| yihQ | -1.33 | 3.72E-04 | 1.98E-03 | down | sulfoquinovosidase |
| yihS | -1.55 | 2.25E-16 | 8.39E-15 | down | sulfoquinovose isomerase |
| yihT | -1.58 | 7.15E-14 | 2.07E-12 | down | 6-deoxy-6-sulfofructose-1-phosphate aldolase |
| yihU | -1.19 | 2.69E-09 | 4.09E-08 | down | 3-sulfolactaldehyde reductase |
| yjiA | -1.54 | 1.28E-20 | 7.66E-19 | down | P-loop guanosine triphosphatase YjiA |
| ytfE | -1.03 | 1.84E-06 | 1.66E-05 | down | iron-sulfur cluster repair protein YtfE |
| adhE | 3.68 | 3.94E-107 | 3.97E-104 | up | fused acetaldehyde-CoA dehydrogenase and iron-dependent alcohol dehydrogenasealdehyde/alcohol dehydrogenase AdhE |
| cgtA | 1.00 | 6.34E-11 | 1.31E-09 | up | |
| csrA | 1.06 | 2.35E-07 | 2.50E-06 | up | carbon storage regulator |

| | | | | | |
|---|---|---|---|---|---|
| deaD | 1.17 | 2.09E-09 | 3.25E-08 | up | ATP-dependent RNA helicase DeaD |
| dtpA | 1.16 | 1.12E-10 | 2.21E-09 | up | dipeptide/tripeptide:H+ symporter DtpA |
| ebgA | 1.12 | 1.09E-17 | 4.71E-16 | up | evolved beta-D-galactosidase subunit alpha |
| ECD_RS01300 | 1.96 | 1.12E-26 | 1.19E-24 | up | |
| ECD_RS01455 | 1.37 | 1.64E-04 | 9.68E-04 | up | |
| ECD_RS03375 | 1.18 | 2.43E-06 | 2.14E-05 | up | |
| ECD_RS03380 | 1.47 | 2.94E-06 | 2.53E-05 | up | |
| ECD_RS04800 | 1.11 | 5.67E-06 | 4.58E-05 | up | |
| ECD_RS04830 | 1.22 | 1.61E-14 | 5.09E-13 | up | |
| ECD_RS05280 | 1.82 | 7.26E-03 | 2.52E-02 | up | |
| ECD_RS05890 | 3.05 | 5.36E-29 | 6.74E-27 | up | |
| ECD_RS08040 | 1.03 | 1.43E-02 | 4.38E-02 | up | |
| ECD_RS08220 | 3.48 | 1.49E-07 | 1.64E-06 | up | |
| ECD_RS08535 | 1.09 | 5.67E-11 | 1.18E-09 | up | |
| ECD_RS08555 | 1.38 | 1.47E-18 | 6.88E-17 | up | |
| ECD_RS08875 | 1.52 | 3.63E-10 | 6.41E-09 | up | |
| ECD_RS09355 | 1.03 | 3.53E-02 | 9.04E-02 | up | |
| ECD_RS09405 | 2.55 | 1.50E-57 | 6.70E-55 | up | |
| ECD_RS10085 | 1.06 | 1.85E-13 | 5.16E-12 | up | |
| ECD_RS10735 | 1.36 | 2.51E-06 | 2.20E-05 | up | |
| ECD_RS13000 | 1.06 | 2.39E-08 | 3.02E-07 | up | |
| ECD_RS14635 | 1.15 | 4.34E-05 | 2.98E-04 | up | |
| ECD_RS15030 | 1.10 | 3.12E-10 | 5.59E-09 | up | |
| ECD_RS16145 | 1.04 | 1.31E-02 | 4.05E-02 | up | |
| ECD_RS19835 | 1.41 | 1.42E-08 | 1.87E-07 | up | |
| ECD_RS20480 | 1.01 | 5.23E-11 | 1.10E-09 | up | |
| ECD_RS22720 | 1.98 | 2.38E-02 | 6.67E-02 | up | |

| | | | | | |
|---|---|---|---|---|---|
| ECD_RS24790 | 2.39 | 1.07E-03 | 4.88E-03 | up | |
| ECD_RS25485 | 2.12 | 2.82E-10 | 5.08E-09 | up | |
| ECD_RS25915 | 1.22 | 6.71E-08 | 7.81E-07 | up | |
| ecnA | 4.14 | 5.40E-03 | NA | up | entericidin A lipoprotein, antidote to entericidin B |
| emrE | 1.05 | 7.78E-03 | 2.66E-02 | up | multidrug/betaine/choline efflux transporter EmrE |
| flhC | 1.21 | 5.14E-10 | 8.80E-09 | up | DNA-binding transcriptional dual regulator FlhC |
| flhD | 1.18 | 8.08E-07 | 7.82E-06 | up | DNA-binding transcriptional dual regulator FlhD |
| folA | 1.27 | 2.71E-09 | 4.09E-08 | up | dihydrofolate reductase |
| fruA | 3.78 | 8.26E-105 | 6.65E-102 | up | fructose-specific PTS multiphosphoryl transfer protein FruA |
| fruB | 3.88 | 2.37E-92 | 1.59E-89 | up | fructose-specific PTS multiphosphoryl transfer protein FruB |
| fruK | 3.93 | 5.82E-118 | 1.17E-114 | up | 1-phosphofructokinase |
| gpmM | 1.24 | 8.94E-19 | 4.33E-17 | up | 2,3-bisphosphoglycerate-independent phosphoglycerate mutase |
| grcA | 1.89 | 3.51E-28 | 4.15E-26 | up | stress-induced alternate pyruvate formate-lyase subunit |
| gss | 1.00 | 8.32E-20 | 4.65E-18 | up | fused glutathionylspermidine amidase / glutathionylspermidine synthetase |
| ilvC | 1.59 | 1.72E-15 | 5.76E-14 | up | ketol-acid reductoisomerase (NADP+) |
| infA | 1.27 | 1.52E-06 | 1.38E-05 | up | translation initiation factor IF-1 |
| ldhA | 2.00 | 1.09E-23 | 8.96E-22 | up | D-lactate dehydrogenase |
| malP | 1.37 | 1.27E-21 | 8.82E-20 | up | maltodextrin phosphorylase |
| malQ | 1.20 | 4.35E-13 | 1.16E-11 | up | 4-alpha-glucanotransferase |
| malS | 1.39 | 7.81E-21 | 4.91E-19 | up | alpha-amylase |
| malZ | 1.07 | 2.93E-14 | 9.06E-13 | up | maltodextrin glucosidase |
| manX | 2.41 | 3.04E-38 | 7.19E-36 | up | mannose-specific PTS enzyme IIAB component |
| manY | 2.32 | 3.00E-34 | 5.25E-32 | up | mannose-specific PTS enzyme IIC component |
| manZ | 2.32 | 5.55E-37 | 1.24E-34 | up | mannose-specific PTS enzyme IID component |
| maoP | 1.08 | 5.88E-12 | 1.38E-10 | up | macrodomain Ori protein |
| mgrB | 1.23 | 2.60E-05 | 1.88E-04 | up | PhoQ kinase inhibitor |
| mqo | 1.69 | 2.56E-35 | 4.90E-33 | up | malate:quinone oxidoreductase |

| | | | | | |
|---|---|---|---|---|---|
| mtlD | 1.14 | 1.12E-20 | 6.91E-19 | up | mannitol-1-phosphate 5-dehydrogenase |
| mtlR | 1.24 | 3.15E-11 | 6.81E-10 | up | transcriptional repressor MtlR |
| ndh | 3.64 | 1.42E-65 | 7.13E-63 | up | NADH:quinone oxidoreductase II |
| ndk | 1.06 | 1.16E-05 | 8.95E-05 | up | nucleoside diphosphate kinase |
| pdhR | 1.22 | 2.33E-11 | 5.19E-10 | up | DNA-binding transcriptional dual regulator PdhR |
| pitA | 1.03 | 2.74E-10 | 4.98E-09 | up | metal phosphate:H+ symporter PitA |
| plaP | 1.64 | 4.29E-26 | 4.15E-24 | up | putrescine:H+ symporter PlaP |
| potF | 1.26 | 1.89E-06 | 1.69E-05 | up | putrescine ABC transporter periplasmic binding protein |
| ptsG | 2.25 | 2.74E-52 | 1.00E-49 | up | glucose-specific PTS enzyme IIBC component |
| pyrF | 1.17 | 3.33E-17 | 1.37E-15 | up | orotidine-5'-phosphate decarboxylase |
| rhlE | 1.16 | 3.62E-19 | 1.82E-17 | up | ATP-dependent RNA helicase RhlE |
| rlmG | 1.19 | 7.03E-11 | 1.44E-09 | up | 23S rRNA m2G1835 methyltransferase |
| rlmI | 1.09 | 5.54E-13 | 1.43E-11 | up | 23S rRNA m5C1962 methyltransferase |
| rph | 1.21 | 4.15E-14 | 1.26E-12 | up | truncated RNase PH |
| rpmA | 1.06 | 6.28E-12 | 1.45E-10 | up | 5S ribosomal subunit protein L27 |
| rpmG | 1.72 | 6.15E-07 | 6.18E-06 | up | 5S ribosomal subunit protein L33 |
| rpmH | 1.74 | 1.97E-08 | 2.54E-07 | up | 5S ribosomal subunit protein L34 |
| rpsT | 1.26 | 2.75E-10 | 4.98E-09 | up | 3S ribosomal subunit protein S2 |
| rpsU | 1.76 | 7.12E-15 | 2.31E-13 | up | 3S ribosomal subunit protein S21 |
| rraB | 1.12 | 1.19E-10 | 2.32E-09 | up | ribonuclease E inhibitor protein B |
| rrf_3 | 1.08 | 1.68E-03 | 7.20E-03 | up | |
| rrf_4 | 1.52 | 1.23E-03 | 5.47E-03 | up | |
| sad | 1.16 | 1.23E-18 | 5.81E-17 | up | succinate semialdehyde dehydrogenase (NAD(P)(+)) Sad |
| speA | 1.09 | 5.01E-10 | 8.61E-09 | up | biosynthetic arginine decarboxylase |
| speC | 1.54 | 5.33E-12 | 1.28E-10 | up | constitutive ornithine decarboxylase |
| treB | 1.79 | 1.64E-44 | 5.06E-42 | up | trehalose-specific PTS enzyme IIBC component |
| treC | 1.39 | 1.88E-18 | 8.71E-17 | up | trehalose-6-phosphate hydrolase |

| | | | | | |
|---|---|---|---|---|---|
| uraA | 1.15 | 3.90E-20 | 2.28E-18 | up | uracil:H+ symporter UraA |
| yciH | 1.57 | 1.90E-08 | 2.47E-07 | up | putative translation factor |
| ydfZ | 1.66 | 1.26E-03 | 5.57E-03 | up | putative selenoprotein YdfZ |
| yeiP | 1.23 | 4.66E-13 | 1.23E-11 | up | elongation factor P-like protein YeiP |
| yidC | 1.00 | 3.88E-10 | 6.79E-09 | up | membrane protein insertase YidC |
| yidD | 1.16 | 8.88E-14 | 2.55E-12 | up | membrane protein insertion efficiency factor |
| yqgB | 1.76 | 6.77E-05 | 4.46E-04 | up | acid stress response protein YqgB |

2

**Table S4 Proteins identified in pull-down assay**

| Accession | Protein | -10lgP | Coverage(%) | | | | | | Description |
|---|---|---|---|---|---|---|---|---|---|
| | | | Control | | | TseL | | | |
| | | | 1 | 2 | 3 | 1 | 2 | 3 | |
| P0C054 | IBPA | 225.43 | 0 | 0 | 0 | 53 | 57 | 58 | Small heat shock protein IbpA |
| P76539 | YPEA | 253.1 | 0 | 0 | 0 | 58 | 58 | 58 | Acetyltransferase YpeA |
| P65294 | YGDR | 123.01 | 0 | 0 | 0 | 56 | 56 | 12 | Uncharacterized lipoprotein YgdR |
| P0ADA7 | OSMB | 36.25 | 0 | 0 | 0 | 56 | 56 | 56 | Osmotically-inducible lipoprotein B |
| P0C058 | IBPB | 141.94 | 0 | 0 | 0 | 42 | 42 | 42 | Small heat shock protein IbpB |
| P0AFW4 | RNK | 100.76 | 0 | 0 | 0 | 40 | 10 | 26 | Regulator of nucleoside diphosphate kinase |
| P0ACJ0 | LRP | 115.86 | 0 | 0 | 0 | 12 | 12 | 21 | Leucine-responsive regulatory protein |
| P62620 | ISPG | 180.04 | 0 | 0 | 0 | 21 | 25 | 14 | 4-hydroxy-3-methylbut-2-en-1-yl diphosphate synthase |
| P52644 | HSLJ | 106.31 | 0 | 0 | 0 | 29 | 14 | 11 | Heat shock protein HslJ |
| P0A6E1 | AROL | 74.23 | 0 | 0 | 0 | 16 | 17 | 16 | Shikimate kinase 2 |
| P76558 | MAO2 | 298.55 | 0 | 0 | 0 | 25 | 27 | 19 | NADP-dependent malic enzyme |
| P13035 | GLPD | 163.02 | 0 | 0 | 0 | 22 | 12 | 10 | Aerobic glycerol-3-phosphate dehydrogenase |
| P76537 | YFEY | 137.16 | 0 | 0 | 0 | 22 | 21 | 28 | Uncharacterized protein YfeY |
| P0A9R7 | FTSE | 118.97 | 0 | 0 | 0 | 24 | 15 | 15 | Cell division ATP-binding protein FtsE |
| P56258 | WECF | 157.91 | 0 | 0 | 0 | 19 | 11 | 19 | 4-alpha-L-fucosyltransferase |
| P77475 | YQAB | 110.01 | 0 | 0 | 0 | 22 | 11 | 22 | Fructose-1-phosphate phosphatase YqaB |
| P37440 | UCPA | 143.08 | 0 | 0 | 0 | 21 | 11 | 17 | Oxidoreductase UcpA |
| P0AB26 | YCEB | 93 | 0 | 0 | 0 | 12 | 12 | 20 | Uncharacterized lipoprotein YceB |
| P0AAI5 | FABF | 78.5 | 0 | 0 | 0 | 12 | 8 | 13 | 3-oxoacyl-[acyl-carrier-protein] synthase 2 |
| Q47710 | YQJK | 67.17 | 0 | 0 | 0 | 18 | 18 | 11 | Uncharacterized protein YqjK |
| P0A8K1 | PSD | 113.6 | 0 | 0 | 0 | 13 | 4 | 12 | Phosphatidylserine decarboxylase proenzyme |
| P40710 | NLPE | 90.83 | 0 | 0 | 0 | 17 | 17 | 8 | Lipoprotein NlpE |

| | | | | | | | | | |
|---|---|---|---|---|---|---|---|---|---|
| P64493 | YOAF | 90.68 | 0 | 0 | 0 | 17 | 17 | 17 | Uncharacterized protein YoaF |
| P0A959 | ALAA | 136.97 | 0 | 0 | 0 | 16 | 7 | 7 | Glutamate-pyruvate aminotransferase AlaA |
| P0AFP6 | YBGI | 102.2 | 0 | 0 | 0 | 8 | 15 | 15 | UPF0135 protein YbgI |
| P00962 | SYQ | 143.56 | 0 | 0 | 0 | 14 | 7 | 9 | Glutamine--tRNA ligase |
| P30014 | RNT | 94.88 | 0 | 0 | 0 | 14 | 6 | 6 | Ribonuclease T |
| P03004 | DNAA | 113.55 | 0 | 0 | 0 | 4 | 10 | 7 | Chromosomal replication initiator protein DnaA |
| P0A935 | MLTA | 108.8 | 0 | 0 | 0 | 13 | 13 | 13 | Membrane-bound lytic murein transglycosylase A |
| P29745 | PEPT | 118.72 | 0 | 0 | 0 | 6 | 10 | 6 | Peptidase T |
| P0ADZ7 | YAJC | 78.89 | 0 | 0 | 0 | 12 | 12 | 12 | UPF0092 membrane protein YajC |
| P21177 | FADB | 144.72 | 0 | 0 | 0 | 7 | 5 | 5 | Fatty acid oxidation complex subunit alpha |
| P0A9D8 | DAPD | 121.27 | 0 | 0 | 0 | 11 | 6 | 11 | 2 3 4 5-tetrahydropyridine-2 6-dicarboxylate N-succinyltransferase |
| P0A8C1 | YBJQ | 61.41 | 0 | 0 | 0 | 11 | 11 | 11 | UPF0145 protein YbjQ |
| P06992 | RSMA | 96.77 | 0 | 0 | 0 | 4 | 4 | 7 | Ribosomal RNA small subunit methyltransferase A |
| P77434 | ALAC | 91.44 | 0 | 0 | 0 | 8 | 7 | 9 | Glutamate-pyruvate aminotransferase AlaC |
| P0AGG8 | TLDD | 90.14 | 0 | 0 | 0 | 10 | 5 | 5 | Protein TldD |
| P04994 | EX7L | 74.93 | 0 | 0 | 0 | 5 | 5 | 8 | Exodeoxyribonuclease 7 large subunit |
| P0AFB1 | NLPI | 74.56 | 0 | 0 | 0 | 8 | 8 | 5 | Lipoprotein NlpI |
| P0AFP4 | YBBO | 69.54 | 0 | 0 | 0 | 8 | 8 | 8 | Uncharacterized oxidoreductase YbbO |
| P75915 | YCDY | 50.29 | 0 | 0 | 0 | 8 | 8 | 8 | Chaperone protein YcdY |
| P0A8M0 | SYN | 81.96 | 0 | 0 | 0 | 5 | 7 | 5 | Asparagine--tRNA ligase |
| P0AE01 | TRMJ | 68.88 | 0 | 0 | 0 | 7 | 7 | 7 | tRNA (cytidine/uridine-2'-O-)-methyltransferase TrmJ |
| P0ACG8 | HSLR | 36.34 | 0 | 0 | 0 | 6 | 6 | 6 | Heat shock protein 15 |
| P31808 | YCIK | 67.32 | 0 | 0 | 0 | 5 | 5 | 5 | Uncharacterized oxidoreductase YciK |
| P0A6T9 | GCSH | 35.15 | 0 | 0 | 0 | 5 | 5 | 5 | Glycine cleavage system H protein |
| P0AGI8 | TRKA | 72.01 | 0 | 0 | 0 | 4 | 4 | 4 | Trk system potassium uptake protein TrkA |
| P66817 | DIAA | 25.9 | 0 | 0 | 0 | 4 | 4 | 4 | DnaA initiator-associating protein DiaA |

Table S5 List of *Pseudomonas* isolates encoding TseL$^{PA}$ protein.

| Subject Replicon Name | Isolation | YEAR | Bioproject | Locus Tag | % Identity | Mismatches |
|---|---|---|---|---|---|---|
| Pseudomonas aeruginosa PACS2 | clinical isolate | 2006 | PRJNA325248 | A0K_RS09885 | 95.6 | 32 |
| Pseudomonas aeruginosa PA7 | clinical isolate | 2007 | PRJNA776240 | PSPA7_3943 | 96.3 | 19 |
| Pseudomonas aeruginosa B136-33 | clinical isolate | 2013 | PRJNA185969 | G655_18235 | 97.7 | 17 |
| Pseudomonas aeruginosa PAK | clinical isolate | 2013 | PRJNA611939 | PAK_03963 | 95.6 | 32 |
| Pseudomonas aeruginosa VRFPA03 | clinical isolate | 2013 | PRJNA209587 | M770_11185 | 96.0 | 30 |
| Pseudomonas aeruginosa CF18 | clinical isolate | 2013 | PRJNA219850 | Q002_03042 | 95.6 | 32 |
| Pseudomonas aeruginosa BL07 | clinical isolate | 2013 | PRJNA219910 | Q061_00374 | 97.1 | 21 |
| Pseudomonas aeruginosa BWHPSA001 | clinical isolate | 2013 | PRJNA219862 | Q014_03487 | 97.2 | 20 |
| Pseudomonas aeruginosa BWHPSA009 | clinical isolate | 2013 | PRJNA219870 | Q022_03762 | 97.2 | 20 |
| Pseudomonas aeruginosa BWHPSA015 | clinical isolate | 2013 | PRJNA219876 | Q028_03781 | 97.2 | 20 |
| Pseudomonas aeruginosa C40 | environmental isolate | 2013 | PRJNA219936 | Q087_03316 | 97.2 | 20 |
| Pseudomonas aeruginosa BL04 | clinical isolate | 2013 | PRJNA219907 | Q058_02985 | 98.9 | 8 |
| Pseudomonas aeruginosa VRFPA05 | clinical isolate | 2013 | PRJNA222642 | T266_RS41500 | 97.3 | 20 |
| Pseudomonas aeruginosa PA1R | lab strain | 2013 | PRJNA185336 | PA1R_gp4992 | 97.3 | 14 |
| Pseudomonas aeruginosa JD318 | clinical isolate | 2013 | PRJNA198782 | DZ35_RS11625 | 97.3 | 19 |
| Pseudomonas aeruginosa MW3a | environmental isolate | 2014 | PRJNA235238 | AK48_RS0116405 | 97.2 | 20 |
| Pseudomonas aeruginosa 3574 | clinical isolate | 2014 | PRJNA234685 | AJ61_00115 | 95.3 | 34 |
| Pseudomonas aeruginosa 3577 | clinical isolate | 2014 | PRJNA234688 | AJ64_03163 | 95.6 | 32 |
| Pseudomonas aeruginosa BWH055 | clinical isolate | 2014 | PRJNA227538 | V556_03046 | 97.2 | 20 |
| Pseudomonas aeruginosa BWH054 | clinical isolate | 2014 | PRJNA227537 | V555_03852 | 97.9 | 16 |
| Pseudomonas aeruginosa PUPa3 | environmental isolate | 2014 | PRJNA325248 | DQ20_16455 | 97.2 | 20 |
| Pseudomonas aeruginosa JMM | environmental isolate | 2014 | PRJNA245482 | RLJV_05320 | 97.1 | 22 |
| Pseudomonas aeruginosa MRSN18971 | clinical isolate | 2014 | PRJNA273956 | CH80_RS10345 | 97.2 | 20 |
| Pseudomonas aeruginosa VRFPA01 | clinical isolate | 2014 | PRJNA183455 | G039_36399 | 96.5 | 21 |

| | | | | | | |
|---|---|---|---|---|---|---|
| Pseudomonas aeruginosa P2-L230/95 | clinical isolate | 2014 | PRJNA243996 | D480_RS02825 | 95.6 | 32 |
| Pseudomonas aeruginosa P7-L633/96 | clinical isolate | 2014 | PRJNA243999 | D407_RS01445 | 96.0 | 30 |
| Pseudomonas aeruginosa AZPAE14975 | clinical isolate | 2014 | PRJNA264310 | NS90_RS11255 | 94.9 | 37 |
| Pseudomonas aeruginosa AZPAE14908 | clinical isolate | 2014 | PRJNA264310 | NS24_RS18455 | 95.3 | 34 |
| Pseudomonas aeruginosa AZPAE14921 | clinical isolate | 2014 | PRJNA264310 | NS37_RS28285 | 95.3 | 34 |
| Pseudomonas aeruginosa AZPAE14931 | clinical isolate | 2014 | PRJNA264310 | NS47_RS17550 | 95.3 | 34 |
| Pseudomonas aeruginosa AZPAE14941 | clinical isolate | 2014 | PRJNA264310 | NS57_RS28725 | 95.3 | 34 |
| Pseudomonas aeruginosa AZPAE14942 | clinical isolate | 2014 | PRJNA264310 | NS58_RS22420 | 95.3 | 34 |
| Pseudomonas aeruginosa AZPAE15061 | clinical isolate | 2014 | PRJNA264310 | NT77_RS29460 | 95.3 | 34 |
| Pseudomonas aeruginosa AZPAE15064 | clinical isolate | 2014 | PRJNA264310 | NT80_RS14575 | 95.3 | 34 |
| Pseudomonas aeruginosa AZPAE12140 | clinical isolate | 2014 | PRJNA264310 | NQ02_RS16725 | 95.4 | 33 |
| Pseudomonas aeruginosa AZPAE12413 | clinical isolate | 2014 | PRJNA264310 | NQ22_RS22660 | 95.4 | 33 |
| Pseudomonas aeruginosa AZPAE12414 | clinical isolate | 2014 | PRJNA264310 | NQ23_RS24705 | 95.4 | 33 |
| Pseudomonas aeruginosa AZPAE12415 | clinical isolate | 2014 | PRJNA264310 | NQ24_RS18450 | 95.4 | 33 |
| Pseudomonas aeruginosa AZPAE14533 | clinical isolate | 2014 | PRJNA264310 | NQ76_RS03165 | 95.4 | 33 |
| Pseudomonas aeruginosa AZPAE14825 | clinical isolate | 2014 | PRJNA264310 | NR43_RS02070 | 95.4 | 33 |
| Pseudomonas aeruginosa AZPAE14977 | clinical isolate | 2014 | PRJNA264310 | NS92_RS11155 | 95.4 | 33 |
| Pseudomonas aeruginosa AZPAE14441 | clinical isolate | 2014 | PRJNA264310 | NQ67_RS00595 | 95.6 | 32 |
| Pseudomonas aeruginosa AZPAE14895 | clinical isolate | 2014 | PRJNA264310 | NS12_RS04760 | 95.6 | 32 |
| Pseudomonas aeruginosa AZPAE14903 | clinical isolate | 2014 | PRJNA264310 | NS19_RS23720 | 95.6 | 32 |
| Pseudomonas aeruginosa AZPAE15042 | clinical isolate | 2014 | PRJNA264310 | NT58_RS08565 | 95.6 | 32 |
| Pseudomonas aeruginosa AZPAE15042 | clinical isolate | 2014 | PRJNA264310 | IHMA87_RS19400 | 95.6 | 32 |
| Pseudomonas aeruginosa AZPAE14967 | clinical isolate | 2014 | PRJNA264310 | NS82_RS22370 | 95.7 | 31 |
| Pseudomonas aeruginosa AZPAE14919 | clinical isolate | 2014 | PRJNA264310 | NS35_RS05480 | 95.8 | 30 |
| Pseudomonas aeruginosa AZPAE13848 | clinical isolate | 2014 | PRJNA264310 | NQ35_RS09745 | 96.0 | 30 |
| Pseudomonas aeruginosa AZPAE14398 | clinical isolate | 2014 | PRJNA264310 | NQ59_RS26225 | 96.0 | 29 |
| Pseudomonas aeruginosa AZPAE14858 | clinical isolate | 2014 | PRJNA264310 | NR74_RS19255 | 96.0 | 29 |

| | | | | | | |
|---|---|---|---|---|---|---|
| Pseudomonas aeruginosa AZPAE14889 | clinical isolate | 2014 | PRJNA264310 | NS05_RS16570 | 96.0 | 29 |
| Pseudomonas aeruginosa AZPAE14911 | clinical isolate | 2014 | PRJNA264310 | NS27_RS27165 | 96.0 | 29 |
| Pseudomonas aeruginosa AZPAE14959 | clinical isolate | 2014 | PRJNA264310 | NS75_RS21180 | 96.0 | 29 |
| Pseudomonas aeruginosa AZPAE14962 | clinical isolate | 2014 | PRJNA264310 | NS78_RS30475 | 96.0 | 29 |
| Pseudomonas aeruginosa AZPAE14991 | clinical isolate | 2014 | PRJNA264310 | NT06_RS25235 | 96.0 | 30 |
| Pseudomonas aeruginosa AZPAE15008 | clinical isolate | 2014 | PRJNA264310 | NT23_RS12975 | 96.0 | 29 |
| Pseudomonas aeruginosa AZPAE14901 | clinical isolate | 2014 | PRJNA264310 | NS17_RS20815 | 96.4 | 26 |
| Pseudomonas aeruginosa AZPAE14877 | clinical isolate | 2014 | PRJNA264310 | NR93_RS14475 | 96.9 | 22 |
| Pseudomonas aeruginosa AZPAE14828 | clinical isolate | 2014 | PRJNA264310 | NR46_RS26675 | 97.1 | 21 |
| Pseudomonas aeruginosa AZPAE15052 | clinical isolate | 2014 | PRJNA264310 | NT68_RS19820 | 97.1 | 21 |
| Pseudomonas aeruginosa AZPAE13872 | clinical isolate | 2014 | PRJNA264310 | NQ43_RS14700 | 97.2 | 20 |
| Pseudomonas aeruginosa AZPAE14373 | clinical isolate | 2014 | PRJNA264310 | NQ52_RS07665 | 97.2 | 20 |
| Pseudomonas aeruginosa AZPAE14687 | clinical isolate | 2014 | PRJNA264310 | NQ84_RS23500 | 97.2 | 20 |
| Pseudomonas aeruginosa AZPAE14818 | clinical isolate | 2014 | PRJNA264310 | NR37_RS01020 | 97.2 | 20 |
| Pseudomonas aeruginosa AZPAE14873 | clinical isolate | 2014 | PRJNA264310 | NR89_RS04180 | 97.2 | 20 |
| Pseudomonas aeruginosa AZPAE14876 | clinical isolate | 2014 | PRJNA264310 | NR92_RS07120 | 97.2 | 20 |
| Pseudomonas aeruginosa AZPAE14910 | clinical isolate | 2014 | PRJNA264310 | NS26_RS08085 | 97.2 | 20 |
| Pseudomonas aeruginosa AZPAE14913 | clinical isolate | 2014 | PRJNA264310 | NS29_RS07550 | 97.2 | 20 |
| Pseudomonas aeruginosa AZPAE14925 | clinical isolate | 2014 | PRJNA264310 | NS41_RS15875 | 97.2 | 20 |
| Pseudomonas aeruginosa AZPAE14939 | clinical isolate | 2014 | PRJNA264310 | NS55_RS20820 | 97.2 | 20 |
| Pseudomonas aeruginosa AZPAE14947 | clinical isolate | 2014 | PRJNA264310 | NS63_RS25415 | 97.2 | 20 |
| Pseudomonas aeruginosa AZPAE14982 | clinical isolate | 2014 | PRJNA264310 | NS97_RS28920 | 97.2 | 20 |
| Pseudomonas aeruginosa AZPAE14960 | clinical isolate | 2014 | PRJNA264310 | NS76_RS21280 | 99.2 | 6 |
| Pseudomonas aeruginosa MRSN 20176 | clinical isolate | 2015 | PRJNA273956 | TN45_RS09060 | 96.0 | 30 |
| Pseudomonas aeruginosa MRSN 20176 | clinical isolate | 2015 | PRJNA273956 | TN45_RS09070 | 96.0 | 30 |
| Pseudomonas aeruginosa 1046_PAER | clinical isolate | 2015 | PRJNA267549 | ADL53_RS17860 | 95.0 | 36 |
| Pseudomonas aeruginosa 719_PAER | clinical isolate | 2015 | PRJNA267549 | ADE99_RS25160 | 95.6 | 32 |

| | | | | | | |
|---|---|---|---|---|---|---|
| Pseudomonas aeruginosa 720_PAER | clinical isolate | 2015 | PRJNA267549 | ADF56_RS11605 | 95.6 | 32 |
| Pseudomonas aeruginosa 577_PAER | clinical isolate | 2015 | PRJNA267549 | ADD94_RS08760 | 96.0 | 29 |
| Pseudomonas aeruginosa 681_PAER | clinical isolate | 2015 | PRJNA267549 | ADE81_RS18645 | 97.2 | 20 |
| Pseudomonas aeruginosa 685_PAER | clinical isolate | 2015 | PRJNA267549 | ADF12_RS17530 | 97.2 | 20 |
| Pseudomonas aeruginosa 710_PAER | clinical isolate | 2015 | PRJNA267549 | ADF13_RS09645 | 97.2 | 20 |
| Pseudomonas aeruginosa 367_PAER | clinical isolate | 2015 | PRJNA267549 | ADD70_RS32745 | 98.9 | 8 |
| Pseudomonas aeruginosa F9676 | environmental isolate | 2015 | PRJNA289296 | ADJ52_RS08105 | 97.2 | 20 |
| Pseudomonas aeruginosa EML548 | clinical isolate | 2015 | PRJNA289008 | AOA67_RS18385 | 94.9 | 37 |
| Pseudomonas aeruginosa EML528 | clinical isolate | 2015 | PRJNA288992 | AOA68_RS18065 | 95.3 | 34 |
| Pseudomonas aeruginosa EML545 | clinical isolate | 2015 | PRJNA289003 | AOA64_RS17815 | 96.2 | 27 |
| Pseudomonas aeruginosa PA1RG | environmental isolate | 2015 | PRJNA295473 | AOD73_RS18950 | 95.4 | 33 |
| Pseudomonas aeruginosa ATCC 9027 | clinical isolate | 2015 | PRJNA294638 | AN920_RS22380 | 94.9 | 37 |
| Pseudomonas aeruginosa PASS1 | clinical isolate | 2015 | PRJNA295120 | AOA73_RS15930 | 96.6 | 24 |
| Pseudomonas aeruginosa PASS3 | clinical isolate | 2015 | PRJNA295122 | AOA75_RS11695 | 96.6 | 24 |
| Pseudomonas aeruginosa 14649 | clinical isolate | 2015 | PRJNA297686 | APG03_RS09480 | 97.2 | 20 |
| Pseudomonas aeruginosa 14650 | clinical isolate | 2015 | PRJNA297686 | APG04_RS07415 | 97.2 | 20 |
| Pseudomonas aeruginosa 14672 | clinical isolate | 2015 | PRJNA297686 | APG06_RS06440 | 97.2 | 20 |
| Pseudomonas aeruginosa 14673 | clinical isolate | 2015 | PRJNA297686 | APG07_RS11005 | 97.2 | 20 |
| Pseudomonas aeruginosa ATCC 33351 | clinical isolate | 2015 | PRJNA294638 | AN446_RS22850 | 95.4 | 33 |
| Pseudomonas aeruginosa ATCC 33348 | clinical isolate | 2015 | PRJNA294638 | AN280_RS16125 | 95.6 | 32 |
| Pseudomonas aeruginosa PS1 | clinical isolate | 2015 | PRJNA295426 | EB236_RS26445 | 96.0 | 29 |
| Pseudomonas aeruginosa ATCC 33358 | clinical isolate | 2015 | PRJNA294638 | AN453_RS20730 | 95.6 | 32 |
| Pseudomonas aeruginosa ATCC 33359 | clinical isolate | 2015 | PRJNA294638 | AN454_RS28680 | 96.2 | 27 |
| Pseudomonas aeruginosa ATCC 33362 | clinical isolate | 2015 | PRJNA294638 | AN457_RS13455 | 97.1 | 21 |
| Pseudomonas aeruginosa WH-SGI-V-07420 | clinical isolate | 2015 | PRJNA297679 | AO994_RS02985 | 91.6 | 60 |

| | | | | | | |
|---|---|---|---|---|---|---|
| Pseudomonas aeruginosa WH-SGI-V-07261 | clinical isolate | 2015 | PRJNA297679 | APB27_RS03925 | 95.0 | 36 |
| Pseudomonas aeruginosa WH-SGI-V-07286 | clinical isolate | 2015 | PRJNA297679 | APB44_RS25225 | 95.0 | 36 |
| Pseudomonas aeruginosa WH-SGI-V-07234 | clinical isolate | 2015 | PRJNA297679 | AO940_RS17475 | 95.3 | 34 |
| Pseudomonas aeruginosa WH-SGI-V-07055 | clinical isolate | 2015 | PRJNA297679 | AO882_RS22025 | 95.4 | 33 |
| Pseudomonas aeruginosa WH-SGI-V-07179 | clinical isolate | 2015 | PRJNA297679 | AO917_RS25950 | 95.4 | 33 |
| Pseudomonas aeruginosa WH-SGI-V-07279 | clinical isolate | 2015 | PRJNA297679 | APB38_RS12310 | 95.4 | 33 |
| Pseudomonas aeruginosa WH-SGI-V-07280 | clinical isolate | 2015 | PRJNA297679 | APB39_RS13395 | 95.4 | 33 |
| Pseudomonas aeruginosa WH-SGI-V-07373 | clinical isolate | 2015 | PRJNA297679 | AO958_RS14610 | 95.4 | 32 |
| Pseudomonas aeruginosa WH-SGI-V-07618 | clinical isolate | 2015 | PRJNA297679 | APA44_RS10765 | 95.4 | 33 |
| Pseudomonas aeruginosa WH-SGI-V-07061 | clinical isolate | 2015 | PRJNA297679 | AO894_RS19895 | 95.6 | 32 |
| Pseudomonas aeruginosa WH-SGI-V-07062 | clinical isolate | 2015 | PRJNA297679 | AO895_RS19780 | 95.6 | 32 |
| Pseudomonas aeruginosa WH-SGI-V-07226 | clinical isolate | 2015 | PRJNA297679 | AO932_RS10855 | 95.6 | 32 |
| Pseudomonas aeruginosa WH-SGI-V-07253 | clinical isolate | 2015 | PRJNA297679 | APB20_RS30055 | 95.6 | 32 |

| | | | | | | |
|---|---|---|---|---|---|---|
| Pseudomonas aeruginosa WH-SGI-V-07692 | clinical isolate | 2015 | PRJNA297679 | APA93_RS19720 | 95.6 | 32 |
| Pseudomonas aeruginosa WH-SGI-V-07050 | clinical isolate | 2015 | PRJNA297679 | AO902_RS28745 | 95.7 | 31 |
| Pseudomonas aeruginosa WH-SGI-V-07054 | clinical isolate | 2015 | PRJNA297679 | AO891_RS10615 | 96.0 | 29 |
| Pseudomonas aeruginosa WH-SGI-V-07169 | clinical isolate | 2015 | PRJNA297679 | AO907_RS13415 | 96.0 | 29 |
| Pseudomonas aeruginosa WH-SGI-V-07232 | clinical isolate | 2015 | PRJNA297679 | AO938_RS14915 | 96.0 | 29 |
| Pseudomonas aeruginosa WH-SGI-V-07313 | clinical isolate | 2015 | PRJNA297679 | APB61_RS21680 | 96.0 | 30 |
| Pseudomonas aeruginosa WH-SGI-V-07318 | clinical isolate | 2015 | PRJNA297679 | APB65_RS23975 | 96.0 | 29 |
| Pseudomonas aeruginosa WH-SGI-V-07369 | clinical isolate | 2015 | PRJNA297679 | AO954_RS08860 | 96.0 | 29 |
| Pseudomonas aeruginosa WH-SGI-V-07381 | clinical isolate | 2015 | PRJNA297679 | AO966_RS19025 | 96.0 | 29 |
| Pseudomonas aeruginosa WH-SGI-V-07418 | clinical isolate | 2015 | PRJNA297679 | AO992_RS11600 | 96.0 | 30 |
| Pseudomonas aeruginosa WH-SGI-V-07493 | clinical isolate | 2015 | PRJNA297679 | APA27_RS26665 | 96.0 | 30 |
| Pseudomonas aeruginosa WH-SGI-V-07619 | clinical isolate | 2015 | PRJNA297679 | APA45_RS11045 | 96.0 | 29 |
| Pseudomonas aeruginosa WH-SGI-V-07648 | clinical isolate | 2015 | PRJNA297679 | APA74_RS27465 | 96.0 | 30 |

| | | | | | | |
|---|---|---|---|---|---|---|
| Pseudomonas aeruginosa WH-SGI-V-07680 | clinical isolate | 2015 | PRJNA297679 | APA81_RS11910 | 96.0 | 29 |
| Pseudomonas aeruginosa WH-SGI-V-07696 | clinical isolate | 2015 | PRJNA297679 | APA97_RS26295 | 96.0 | 30 |
| Pseudomonas aeruginosa WH-SGI-V-07314 | clinical isolate | 2015 | PRJNA297679 | APB62_RS11710 | 96.1 | 28 |
| Pseudomonas aeruginosa WH-SGI-V-07376 | clinical isolate | 2015 | PRJNA297679 | AO961_RS20015 | 96.1 | 28 |
| Pseudomonas aeruginosa WH-SGI-V-07252 | clinical isolate | 2015 | PRJNA297679 | APB19_RS02660 | 96.5 | 26 |
| Pseudomonas aeruginosa WH-SGI-V-07064 | clinical isolate | 2015 | PRJNA297679 | AO896_RS02425 | 96.6 | 24 |
| Pseudomonas aeruginosa WH-SGI-V-07072 | clinical isolate | 2015 | PRJNA297679 | AO898_RS02430 | 96.6 | 24 |
| Pseudomonas aeruginosa WH-SGI-V-07370 | clinical isolate | 2015 | PRJNA297679 | AO955_RS12165 | 96.6 | 24 |
| Pseudomonas aeruginosa WH-SGI-V-07426 | clinical isolate | 2015 | PRJNA297679 | APA00_RS20340 | 96.6 | 25 |
| Pseudomonas aeruginosa WH-SGI-V-07165 | clinical isolate | 2015 | PRJNA297679 | AO903_RS02745 | 96.8 | 23 |
| Pseudomonas aeruginosa WH-SGI-V-07422 | clinical isolate | 2015 | PRJNA297679 | AO996_RS09360 | 97.0 | 17 |
| Pseudomonas aeruginosa WH-SGI-V-07187 | clinical isolate | 2015 | PRJNA297679 | AO926_RS03260 | 97.2 | 20 |
| Pseudomonas aeruginosa WH-SGI-V-07372 | clinical isolate | 2015 | PRJNA297679 | AO957_RS00580 | 97.2 | 20 |

| | | | | | | |
|---|---|---|---|---|---|---|
| Pseudomonas aeruginosa WH-SGI-V-07413 | clinical isolate | 2015 | PRJNA297679 | AO987_RS03260 | 97.2 | 20 |
| Pseudomonas aeruginosa WH-SGI-V-07632 | clinical isolate | 2015 | PRJNA297679 | APA58_RS18020 | 97.2 | 20 |
| Pseudomonas aeruginosa WH-SGI-V-07647 | clinical isolate | 2015 | PRJNA297679 | APA73_RS23020 | 97.2 | 20 |
| Pseudomonas aeruginosa WH-SGI-V-07263 | clinical isolate | 2015 | PRJNA297679 | APB28_RS15860 | 97.3 | 20 |
| Pseudomonas aeruginosa WH-SGI-V-07637 | clinical isolate | 2015 | PRJNA297679 | APA63_RS11585 | 97.5 | 19 |
| Pseudomonas aeruginosa WH-SGI-V-07265 | clinical isolate | 2015 | PRJNA297679 | APB29_RS11865 | 97.9 | 16 |
| Pseudomonas aeruginosa WH-SGI-V-07384 | clinical isolate | 2015 | PRJNA297679 | AO969_RS09720 | 97.9 | 16 |
| Pseudomonas aeruginosa WH-SGI-V-07059 | clinical isolate | 2015 | PRJNA297679 | AO901_RS15480 | 98.9 | 8 |
| Pseudomonas aeruginosa WH-SGI-V-07183 | clinical isolate | 2015 | PRJNA297679 | AO922_RS15260 | 98.9 | 8 |
| Pseudomonas aeruginosa WH-SGI-V-07185 | clinical isolate | 2015 | PRJNA297679 | AO924_RS21305 | 98.9 | 8 |
| Pseudomonas aeruginosa SMC4386 | clinical isolate | 2015 | PRJNA305962 | AVR61_RS09290 | 97.1 | 21 |
| Pseudomonas aeruginosa SMC4386_delta_CRISPR_Cas | clinical isolate | 2015 | PRJNA305963 | AVR60_RS08180 | 97.1 | 21 |
| Pseudomonas aeruginosa AU10713 | clinical isolate | 2016 | PRJNA309533 | AW932_RS04740 | 95.3 | 34 |
| Pseudomonas aeruginosa AU17550 | clinical isolate | 2016 | PRJNA309533 | AW933_RS00390 | 95.3 | 34 |
| Pseudomonas aeruginosa AU18132 | clinical isolate | 2016 | PRJNA309533 | AW934_RS11500 | 95.3 | 34 |
| Pseudomonas aeruginosa AU19319 | clinical isolate | 2016 | PRJNA309533 | AW935_RS04735 | 95.3 | 34 |

| | | | | | | |
|---|---|---|---|---|---|---|
| Pseudomonas aeruginosa AU24526 | clinical isolate | 2016 | PRJNA309533 | AW936_RS03965 | 95.3 | 34 |
| Pseudomonas aeruginosa 105777 | clinical isolate | 2016 | PRJNA261239 | LT17_RS05340 | 97.2 | 20 |
| Pseudomonas aeruginosa 105880 | clinical isolate | 2016 | PRJNA261239 | LL05_RS08200 | 96.0 | 29 |
| Pseudomonas aeruginosa PA_D1 | clinical isolate | 2016 | PRJNA294254 | AM599_RS19160 | 96.0 | 29 |
| Pseudomonas aeruginosa PA_D16 | clinical isolate | 2016 | PRJNA294254 | A6695_RS19160 | 96.0 | 29 |
| Pseudomonas aeruginosa PA_D2 | clinical isolate | 2016 | PRJNA294254 | A6681_RS19165 | 96.0 | 29 |
| Pseudomonas aeruginosa PA_D21 | clinical isolate | 2016 | PRJNA294254 | A7329_RS19150 | 96.0 | 29 |
| Pseudomonas aeruginosa PA_D22 | clinical isolate | 2016 | PRJNA294254 | A6701_RS19160 | 96.0 | 29 |
| Pseudomonas aeruginosa PA_D25 | clinical isolate | 2016 | PRJNA294254 | A6704_RS19165 | 96.0 | 29 |
| Pseudomonas aeruginosa PA_D5 | clinical isolate | 2016 | PRJNA294254 | A7331_RS19155 | 96.0 | 29 |
| Pseudomonas aeruginosa PA_D9 | clinical isolate | 2016 | PRJNA294254 | A6688_RS07170 | 96.0 | 29 |
| Pseudomonas aeruginosa PA13SY16 | clinical isolate | 2016 | PRJNA321097 | A7R77_RS01975 | 95.7 | 32 |
| Pseudomonas aeruginosa 9AR3 | clinical isolate | 2016 | PRJNA294726 | AN467_RS10595 | 96.1 | 28 |
| Pseudomonas aeruginosa DN1 | environmental isolate | 2016 | PRJNA341318 | BG483_RS20185 | 96.0 | 30 |
| Pseudomonas aeruginosa 515477 | clinical isolate | 2016 | PRJNA342804 | BH596_RS29660 | 95.4 | 33 |
| Pseudomonas aeruginosa SCH_ABX20 | clinical isolate | 2017 | PRJNA369567 | B0B28_RS11360 | 95.6 | 32 |
| Pseudomonas aeruginosa SCH_ABX10 | clinical isolate | 2017 | PRJNA369567 | B0B17_RS02760 | 97.2 | 20 |
| Pseudomonas aeruginosa SCH_ABX09 | clinical isolate | 2017 | PRJNA369567 | B0B35_RS00590 | 97.2 | 20 |
| Pseudomonas aeruginosa SD9 | clinical isolate | 2017 | PRJNA175101 | C531_RS01100 | 97.2 | 20 |
| Pseudomonas aeruginosa P37 | environmental isolate | 2017 | PRJNA564463 | C532_RS03535 | 97.2 | 20 |
| Pseudomonas aeruginosa P47 | environmental isolate | 2017 | PRJNA175102 | C533_RS03540 | 97.2 | 20 |
| Pseudomonas aeruginosa P49 | environmental isolate | 2017 | PRJNA175102 | C534_RS03545 | 97.2 | 20 |
| Pseudomonas aeruginosa PA_154197 | clinical isolate | 2017 | PRJNA310413 | AXW93_RS19345 | 97.9 | 16 |
| Pseudomonas aeruginosa UQCCR 364724162 AA73 | clinical isolate | 2017 | PRJNA348795 | B1H15_RS13390 | 93.4 | 46 |
| Pseudomonas aeruginosa UQCCR 35166-1996 AC02 | clinical isolate | 2017 | PRJNA348795 | BKN47_RS15245 | 95.3 | 34 |

| | | | | | | |
|---|---|---|---|---|---|---|
| Pseudomonas aeruginosa H5708 | clinical isolate | 2017 | PRJNA253624 | HW07_RS19195 | 95.4 | 33 |
| Pseudomonas aeruginosa UQCCR 393788042 K AB94 | clinical isolate | 2017 | PRJNA348795 | B1H17_RS16565 | 96.0 | 29 |
| Pseudomonas aeruginosa Pb18 | environmental isolate | 2017 | PRJNA320097 | A6748_RS10240 | 95.6 | 32 |
| Pseudomonas aeruginosa M28A1 | environmental isolate | 2017 | PRJNA320098 | A6752_RS04935 | 96.1 | 28 |
| Pseudomonas aeruginosa S53_C01_BS | clinical isolate | 2017 | PRJNA383293 | CAZ05_RS21655 | 96.0 | 29 |
| Pseudomonas aeruginosa S57_C01_BS | clinical isolate | 2017 | PRJNA383293 | CAZ04_RS25720 | 96.0 | 29 |
| Pseudomonas aeruginosa SCH_ABX19 | clinical isolate | 2017 | PRJNA369567 | B0B26_RS28265 | 95.6 | 32 |
| Pseudomonas aeruginosa M26 | clinical isolate | 2017 | PRJNA371681 | B0535_RS12010 | 97.1 | 21 |
| Pseudomonas aeruginosa 2321 | clinical isolate | 2017 | PRJNA342804 | CDC12_RS23535 | 95.6 | 32 |
| Pseudomonas aeruginosa 6005 | clinical isolate | 2017 | PRJNA325248 | CDC22_RS05350 | 96.0 | 29 |
| Pseudomonas aeruginosa PA127 | clinical isolate | 2017 | PRJNA590804 | GNQ21_RS03060 | 95.6 | 32 |
| Pseudomonas aeruginosa Pa84 | clinical isolate | 2017 | PRJNA389181 | CD796_RS20500 | 97.2 | 20 |
| Pseudomonas aeruginosa Pae113 | environmental isolate | 2017 | PRJNA392234 | EQH81_RS11480 | 96.1 | 28 |
| Pseudomonas aeruginosa ATCC BAA-2113 | clinical isolate | 2017 | PRJNA325248 | IPC1581_RS05625 | 95.4 | 33 |
| Pseudomonas aeruginosa ATCC BAA-2109 | clinical isolate | 2017 | PRJNA325248 | IPC1577_RS25085 | 97.2 | 20 |
| Pseudomonas aeruginosa ATCC BAA-2112 | clinical isolate | 2017 | PRJNA325248 | IPC1580_RS21010 | 97.2 | 20 |
| Pseudomonas aeruginosa Env_32 | environmental isolate | 2017 | PRJNA393338 | CF342_RS24275 | 95.2 | 35 |
| Pseudomonas aeruginosa Env_58 | environmental isolate | 2017 | PRJNA393338 | CF344_RS16660 | 97.1 | 21 |
| Pseudomonas aeruginosa BK2 | clinical isolate | 2017 | PRJNA286827 | CIW79_RS19265 | 97.2 | 20 |
| Pseudomonas aeruginosa BK4 | clinical isolate | 2017 | PRJNA287086 | ACG88_RS03280 | 97.2 | 20 |
| Pseudomonas aeruginosa 173 | clinical isolate | 2017 | PRJNA378495、PRJEB5438、PRJNA514442 | B8A38_RS06465 | 95.3 | 34 |

| | | | | | | |
|---|---|---|---|---|---|---|
| Pseudomonas aeruginosa 282 | clinical isolate | 2017 | PRJNA378495、PRJEB5438 | B8B68_RS24045 | 97.2 | 20 |
| Pseudomonas aeruginosa VD171 | clinical isolate | 2017 | PRJNA378495 | B8B71_RS27800 | 97.2 | 20 |
| Pseudomonas aeruginosa VD329 | clinical isolate | 2017 | PRJNA378495 | B8B81_RS21720 | 97.2 | 20 |
| Pseudomonas aeruginosa VD564 | clinical isolate | 2017 | PRJNA378495 | B8B69_RS22705 | 97.2 | 20 |
| Pseudomonas aeruginosa VD609 | clinical isolate | 2017 | PRJNA378495 | B8A61_RS24245 | 97.2 | 20 |
| Pseudomonas aeruginosa VD706 | clinical isolate | 2017 | PRJNA378495 | B8B82_RS22960 | 97.2 | 20 |
| Pseudomonas aeruginosa env210 | environmental isolate | 2017 | PRJNA393338 | CJT97_RS21040 | 95.2 | 35 |
| Pseudomonas aeruginosa S30a | environmental isolate | 2017 | PRJNA393338 | CJU24_RS18395 | 95.3 | 34 |
| Pseudomonas aeruginosa env102 | environmental isolate | 2017 | PRJNA393338 | CJT59_RS06640 | 95.4 | 33 |
| Pseudomonas aeruginosa U5a-2 | environmental isolate | 2017 | PRJNA393338 | CJU28_RS07525 | 95.4 | 33 |
| Pseudomonas aeruginosa env097 | environmental isolate | 2017 | PRJNA393338 | CJT55_RS18100 | 95.6 | 32 |
| Pseudomonas aeruginosa S20b | environmental isolate | 2017 | PRJNA393338 | CJU21_RS08300 | 95.6 | 32 |
| Pseudomonas aeruginosa S25b-2 | environmental isolate | 2017 | PRJNA393338 | CJU23_RS19735 | 95.6 | 32 |
| Pseudomonas aeruginosa env158 | environmental isolate | 2017 | PRJNA393338 | CJT81_RS16570 | 97.2 | 20 |
| Pseudomonas aeruginosa env204 | environmental isolate | 2017 | PRJNA393338 | CJT95_RS03105 | 97.2 | 20 |
| Pseudomonas aeruginosa SJU-S72_1 | environmental isolate | 2017 | PRJNA393338 | CJU48_RS24320 | 97.2 | 20 |
| Pseudomonas aeruginosa SJU-S72_2 | environmental isolate | 2017 | PRJNA393338 | CJU49_RS25460 | 97.2 | 20 |
| Pseudomonas aeruginosa SJU-S72_3 | environmental isolate | 2017 | PRJNA393338 | CJU50_RS24620 | 97.2 | 20 |
| Pseudomonas aeruginosa SJU-W4_1 | environmental isolate | 2017 | PRJNA393338 | CJU54_RS06980 | 97.2 | 20 |
| Pseudomonas aeruginosa SJU-W4_2 | environmental isolate | 2017 | PRJNA393338 | CJU55_RS06545 | 97.2 | 20 |
| Pseudomonas aeruginosa SJU-W4_3 | environmental isolate | 2017 | PRJNA393338 | CJU56_RS03375 | 97.2 | 20 |
| Pseudomonas aeruginosa U5b-2 | environmental isolate | 2017 | PRJNA393338 | CJU29_RS13855 | 97.2 | 20 |
| Pseudomonas aeruginosa W21b-2 | environmental isolate | 2017 | PRJNA393338 | CJU36_RS04925 | 97.2 | 20 |
| Pseudomonas aeruginosa 85 | clinical isolate | 2017 | PRJNA411997、PRJEB5438、PRJNA514442 | CP912_RS23620 | 96.0 | 29 |

| | | | | | | |
|---|---|---|---|---|---|---|
| Pseudomonas aeruginosa isolate 85 | clinical isolate | 2017 | PRJEB5438 | BUR14_RS28970 | 96.6 | 25 |
| Pseudomonas aeruginosa isolate 130 | clinical isolate | 2017 | PRJEB5438 | BUF87_RS00115 | 97.1 | 21 |
| Pseudomonas aeruginosa 140 | clinical isolate | 2017 | PRJNA514442、PRJNA411997、PRJEB5438 | EQ648_RS18960 | 97.2 | 20 |
| Pseudomonas aeruginosa PA34 | clinical isolate | 2017 | PRJNA855568 | PA34_RS12750 | 96.0 | 29 |
| Pseudomonas aeruginosa 12939 | clinical isolate | 2017 | PRJNA415682 | CS058_RS19090 | 97.2 | 20 |
| Pseudomonas aeruginosa PA3 | clinical isolate | 2018 | PRJNA776240 | CEA86_RS27120 | 95.3 | 34 |
| Pseudomonas aeruginosa 192S190811BSL_PA1 | clinical isolate | 2018 | PRJNA325248 | IPC43_RS02675 | 95.4 | 33 |
| Pseudomonas aeruginosa 192S190811BSL_PA3 | clinical isolate | 2018 | PRJNA325248 | IPC45_RS03515 | 95.4 | 33 |
| Pseudomonas aeruginosa PA1 | clinical isolate | 2018 | PRJNA776240 | PA1S_RS18955 | 95.4 | 33 |
| Pseudomonas aeruginosa 197S020911BSL_PA3 | clinical isolate | 2018 | PRJNA325248 | IPC48_RS12035 | 96.0 | 29 |
| Pseudomonas aeruginosa PA4 | clinical isolate | 2018 | PRJNA776240 | CEA87_RS16300 | 97.1 | 21 |
| Pseudomonas aeruginosa 903 | clinical isolate-Veterinary | 2018 | PRJNA297512 | IPC96_RS06640 | 97.1 | 21 |
| Pseudomonas aeruginosa Pa64 | clinical isolate | 2018 | PRJNA855568 | C3L32_RS11330 | 95.3 | 34 |
| Pseudomonas aeruginosa 8488 | clinical isolate | 2018 | PRJNA434136 | C5F84_RS19340 | 95.3 | 34 |
| Pseudomonas aeruginosa AR_0360 | clinical isolate | 2018 | PRJNA316321、PRJNA786742 | CSB97_RS13410 | 95.3 | 34 |
| Pseudomonas aeruginosa AR_0356 | clinical isolate | 2018 | PRJNA316321 | CSB93_RS26645 | 96.8 | 23 |
| Pseudomonas aeruginosa WCHPA075006 | clinical isolate | 2018 | PRJNA415331 | C2I08_RS02230 | 95.4 | 33 |
| Pseudomonas aeruginosa WCHPA075015 | clinical isolate | 2018 | PRJNA415331 | C2I09_RS08440 | 96.0 | 29 |
| Pseudomonas aeruginosa WCHPA075017 | clinical isolate | 2018 | PRJNA415331 | C2I11_RS08440 | 96.0 | 29 |
| Pseudomonas aeruginosa WCHPA075056 | clinical isolate | 2018 | PRJNA415331 | C2I15_RS05640 | 96.1 | 28 |

| | | | | | | |
|---|---|---|---|---|---|---|
| Pseudomonas aeruginosa WCHPA075063 | clinical isolate | 2018 | PRJNA415331 | C2I16_RS06050 | 96.1 | 28 |
| Pseudomonas aeruginosa CLJ1 | clinical isolate | 2018 | PRJNA383797 | CLJ1_RS18680 | 95.3 | 34 |
| Pseudomonas aeruginosa HIAE_PA08 | clinical isolate | 2018 | PRJNA449616 | DB382_RS25010 | 95.8 | 30 |
| Pseudomonas aeruginosa KCJK8014 | clinical isolate-Veterinary | 2018 | PRJNA449112 | DBL08_RS15425 | 96.0 | 29 |
| Pseudomonas aeruginosa KCJK8015 | clinical isolate-Veterinary | 2018 | PRJNA449112 | DBL09_RS25150 | 96.0 | 29 |
| Pseudomonas aeruginosa KCJK8016 | clinical isolate-Veterinary | 2018 | PRJNA449112 | DBL10_RS16520 | 96.0 | 29 |
| Pseudomonas aeruginosa HIAE_PA22 | clinical isolate | 2018 | PRJNA449616 | DB396_RS01800 | 97.1 | 21 |
| Pseudomonas aeruginosa HIAE_PA12 | clinical isolate | 2018 | PRJNA449616 | DB380_RS25090 | 99.7 | 2 |
| Pseudomonas aeruginosa CLJ3 | clinical isolate | 2018 | PRJNA383798 | CLJ3_RS18435 | 95.2 | 35 |
| Pseudomonas aeruginosa JB2 | environmental isolate | 2018 | PRJNA433941 | C5B40_RS14015 | 97.2 | 20 |
| Pseudomonas aeruginosa AR441 | clinical isolate | 2018 | PRJNA316321 | CSC28_RS20710 | 96.8 | 23 |
| Pseudomonas aeruginosa KCRI-379A isolate RDK06_379A | clinical isolate | 2018 | PRJEB26613 | DK345_RS26930 | 95.2 | 35 |
| Pseudomonas aeruginosa KCRI-377 isolate R0007_377 | clinical isolate | 2018 | PRJEB26613 | DK330_RS01050 | 95.8 | 30 |
| Pseudomonas aeruginosa KCRI-164A isolate RDK04_164A | clinical isolate | 2018 | PRJEB26613 | DK311_RS14830 | 96.9 | 23 |
| Pseudomonas aeruginosa KCRI-214 isolate R0003_214 | clinical isolate | 2018 | PRJEB26613 | DK310_RS09255 | 97.1 | 21 |
| Pseudomonas aeruginosa 11987-2-5 | environmental isolate | 2018 | PRJNA473071 | DMC54_RS03070 | 98.9 | 8 |
| Pseudomonas aeruginosa NCTC11450 | clinical isolate | 2018 | PRJEB6403 | DS939_RS00690 | 95.6 | 32 |
| Pseudomonas aeruginosa GER_MD14_1510_Pae_083 | clinical isolate-Veterinary | 2018 | PRJNA433857 | C6A84_RS22860 | 95.3 | 34 |
| Pseudomonas aeruginosa F2-1206 | clinical isolate | 2018 | PRJNA431093 | DT383_RS06265 | 97.2 | 20 |

| | | | | | | |
|---|---|---|---|---|---|---|
| Pseudomonas aeruginosa PA57 | clinical isolate | 2018 | PRJNA431326 | PA57_RS02755 | 89.7 | 74 |
| Pseudomonas aeruginosa PA149 | clinical isolate | 2018 | PRJNA431326 | PA149_RS22320 | 97.2 | 20 |
| Pseudomonas aeruginosa PA171 | clinical isolate | 2018 | PRJNA855568 | PA171_RS07725 | 97.2 | 20 |
| Pseudomonas aeruginosa NCTC7244 | clinical isolate | 2018 | PRJEB6403 | DYC87_RS13210 | 95.0 | 36 |
| Pseudomonas aeruginosa NCTC10730 | clinical isolate | 2018 | PRJEB6403 | DYB99_RS13900 | 96.6 | 24 |
| Pseudomonas aeruginosa AUH-PA120 | clinical isolate | 2018 | PRJNA471224 | DI402_RS04040 | 96.6 | 25 |
| Pseudomonas aeruginosa L25 | environmental isolate | 2018 | PRJNA436965 | D0Z20_RS19190 | 96.1 | 28 |
| Pseudomonas aeruginosa M12 | environmental isolate | 2018 | PRJNA436965 | D0Z21_RS15420 | 96.1 | 28 |
| Pseudomonas aeruginosa W-101 | environmental isolate | 2018 | PRJNA484258 | DX920_RS06395 | 95.6 | 32 |
| Pseudomonas aeruginosa PA_151345 | clinical isolate | 2018 | PRJNA310413 | AXX04_RS08555 | 89.0 | 79 |
| Pseudomonas aeruginosa PA_151908 | clinical isolate | 2018 | PRJNA310413 | AXX00_RS07745 | 96.0 | 29 |
| Pseudomonas aeruginosa PA_152165 | clinical isolate | 2018 | PRJNA310413 | AXW98_RS17435 | 96.0 | 29 |
| Pseudomonas aeruginosa PA_152361 | clinical isolate | 2018 | PRJNA310413 | AXW99_RS08375 | 96.0 | 29 |
| Pseudomonas aeruginosa PA_153543 | clinical isolate | 2018 | PRJNA310413 | AXW97_RS29055 | 96.5 | 24 |
| Pseudomonas aeruginosa PA_153673 | clinical isolate | 2018 | PRJNA310413 | AXW94_RS28500 | 97.2 | 20 |
| Pseudomonas aeruginosa PA_4823 | clinical isolate | 2018 | PRJNA310413 | AXW92_RS15375 | 97.2 | 20 |
| Pseudomonas aeruginosa PA_151970 | clinical isolate | 2018 | PRJNA310413 | AXX02_RS05010 | 99.2 | 6 |
| Pseudomonas aeruginosa ENV-454 | environmental isolate | 2018 | PRJNA244279 | DQG61_RS08950 | 89.7 | 74 |
| Pseudomonas aeruginosa ENV-480 | environmental isolate | 2018 | PRJNA244279 | DQG62_RS10490 | 89.7 | 74 |
| Pseudomonas aeruginosa VET-124 | environmental isolate | 2018 | PRJNA244279 | DQG60_RS20005 | 89.7 | 74 |
| Pseudomonas aeruginosa HUM-250 | clinical isolate | 2018 | PRJNA244279 | DQG63_RS07585 | 93.9 | 36 |
| Pseudomonas aeruginosa ENV-568 | environmental isolate | 2018 | PRJNA244279 | DQG65_RS11755 | 93.9 | 36 |
| Pseudomonas aeruginosa ENV-569 | environmental isolate | 2018 | PRJNA244279 | DQG66_RS19005 | 93.9 | 36 |
| Pseudomonas aeruginosa ENV-570 | environmental isolate | 2018 | PRJNA244279 | DQG64_RS05290 | 93.9 | 36 |
| Pseudomonas aeruginosa ENV-550 | environmental isolate | 2018 | PRJNA244279 | DQG56_RS28545 | 95.2 | 35 |
| Pseudomonas aeruginosa ENV-551 | environmental isolate | 2018 | PRJNA244279 | DQG53_RS15815 | 95.2 | 35 |
| Pseudomonas aeruginosa ENV-552 | environmental isolate | 2018 | PRJNA244279 | DQG55_RS21775 | 95.2 | 35 |

| | | | | | | |
|---|---|---|---|---|---|---|
| Pseudomonas aeruginosa ENV-566 | environmental isolate | 2018 | PRJNA244279 | DQG52_RS07985 | 95.2 | 35 |
| Pseudomonas aeruginosa ENV-567 | environmental isolate | 2018 | PRJNA244279 | DQG54_RS16385 | 95.2 | 35 |
| Pseudomonas aeruginosa VET-73 | environmental isolate | 2018 | PRJNA244279 | DQG51_RS00405 | 95.2 | 35 |
| Pseudomonas aeruginosa HUM-235 | clinical isolate | 2018 | PRJNA244279 | DQI65_RS22105 | 95.3 | 34 |
| Pseudomonas aeruginosa HUM-286 | clinical isolate | 2018 | PRJNA244279 | DQI64_RS22480 | 95.3 | 34 |
| Pseudomonas aeruginosa HUM-289 | clinical isolate | 2018 | PRJNA244279 | DQI66_RS01725 | 95.3 | 34 |
| Pseudomonas aeruginosa HUM-325 | clinical isolate | 2018 | PRJNA244279 | DQI67_RS19515 | 95.3 | 34 |
| Pseudomonas aeruginosa HUM-327-D1 repl1 | clinical isolate | 2018 | PRJNA244279 | DQI61_RS24740 | 95.3 | 34 |
| Pseudomonas aeruginosa HUM-327-D1 repl2 | clinical isolate | 2018 | PRJNA244279 | DQI62_RS09505 | 95.3 | 34 |
| Pseudomonas aeruginosa HUM-330 | clinical isolate | 2018 | PRJNA244279 | DQI60_RS23395 | 95.3 | 34 |
| Pseudomonas aeruginosa HUM-333 | clinical isolate | 2018 | PRJNA244279 | DQI63_RS09705 | 95.3 | 34 |
| Pseudomonas aeruginosa HUM-7 | clinical isolate | 2018 | PRJNA244279 | DQH59_RS11450 | 95.3 | 34 |
| Pseudomonas aeruginosa ENV-681 | environmental isolate | 2018 | PRJNA244279 | DQH65_RS36170 | 95.3 | 34 |
| Pseudomonas aeruginosa VET-121 | environmental isolate | 2018 | PRJNA244279 | DQG48_RS09435 | 95.3 | 34 |
| Pseudomonas aeruginosa HUM-294 | clinical isolate | 2018 | PRJNA244279 | DQH08_RS11225 | 95.4 | 33 |
| Pseudomonas aeruginosa HUM-336 | clinical isolate | 2018 | PRJNA244279 | DQH12_RS01975 | 95.4 | 33 |
| Pseudomonas aeruginosa VET-33 | environmental isolate | 2018 | PRJNA244279 | DQI78_RS13100 | 95.4 | 33 |
| Pseudomonas aeruginosa VET-36 | environmental isolate | 2018 | PRJNA244279 | DQI76_RS18785 | 95.4 | 33 |
| Pseudomonas aeruginosa VET-56 | environmental isolate | 2018 | PRJNA244279 | DQH11_RS09490 | 95.4 | 33 |
| Pseudomonas aeruginosa VET-61 | environmental isolate | 2018 | PRJNA244279 | DQH09_RS00690 | 95.4 | 33 |
| Pseudomonas aeruginosa HUM-331 | clinical isolate | 2018 | PRJNA244279 | DQH07_RS01010 | 95.6 | 32 |
| Pseudomonas aeruginosa HUM-242 | clinical isolate | 2018 | PRJNA244279 | DQG57_RS29010 | 95.7 | 31 |
| Pseudomonas aeruginosa HUM-258 | clinical isolate | 2018 | PRJNA244279 | DQH02_RS00495 | 95.7 | 32 |
| Pseudomonas aeruginosa HUM-257 | clinical isolate | 2018 | PRJNA244279 | DQH52_RS19505 | 96.1 | 28 |
| Pseudomonas aeruginosa HUM-261 | clinical isolate | 2018 | PRJNA244279 | DQI03_RS17035 | 96.9 | 23 |

| | | | | | | |
|---|---|---|---|---|---|---|
| Pseudomonas aeruginosa VET-64 | environmental isolate | 2018 | PRJNA244279 | DQH10_RS26150 | 97.0 | 18 |
| Pseudomonas aeruginosa VET-39-D2 | environmental isolate | 2018 | PRJNA244279 | DQH55_RS08545 | 97.1 | 21 |
| Pseudomonas aeruginosa VET-44 | environmental isolate | 2018 | PRJNA244279 | DQH56_RS19500 | 97.1 | 21 |
| Pseudomonas aeruginosa VET-49 | environmental isolate | 2018 | PRJNA244279 | DQH53_RS20365 | 97.1 | 21 |
| Pseudomonas aeruginosa VET-77 | environmental isolate | 2018 | PRJNA244279 | DQH54_RS01035 | 97.1 | 21 |
| Pseudomonas aeruginosa HUM-276 | clinical isolate | 2018 | PRJNA244279 | DQI19_RS13630 | 97.2 | 20 |
| Pseudomonas aeruginosa HUM-374 | clinical isolate | 2018 | PRJNA244279 | DQI18_RS14240 | 97.2 | 20 |
| Pseudomonas aeruginosa ENV-208 | environmental isolate | 2018 | PRJNA244279 | DQG83_RS03895 | 97.2 | 20 |
| Pseudomonas aeruginosa VET-35 | environmental isolate | 2018 | PRJNA244279 | DQI20_RS30785 | 97.2 | 20 |
| Pseudomonas aeruginosa VET-34 | environmental isolate | 2018 | PRJNA244279 | DQI77_RS29395 | 97.4 | 13 |
| Pseudomonas aeruginosa 55AA, isolate PAK | clinical isolate | 2018 | PRJNA325248 | IPC83_RS22650 | 95.6 | 32 |
| Pseudomonas aeruginosa F69A, isolate IST27 | clinical isolate | 2018 | PRJNA325248 | IPC84_RS16100 | 95.7 | 32 |
| Pseudomonas aeruginosa F69A, isolate IST27N | clinical isolate | 2018 | PRJNA325248 | IPC85_RS20170 | 95.7 | 32 |
| Pseudomonas aeruginosa ER06896 | clinical isolate | 2018 | PRJNA400231 | CKU30_RS17825 | 96.6 | 25 |
| Pseudomonas aeruginosa PS00100 | environmental isolate | 2018 | PRJNA400231 | CKQ82_RS24440 | 96.6 | 25 |
| Pseudomonas aeruginosa AUS232 | clinical isolate | 2018 | PRJNA325248 | IPC258_RS05225 | 89.7 | 74 |
| Pseudomonas aeruginosa DUN-004 | clinical isolate | 2018 | PRJNA325248 | IPC1001_RS24880 | 89.7 | 74 |
| Pseudomonas aeruginosa AUS165 | clinical isolate | 2018 | PRJNA325248 | IPC404_RS04010 | 94.9 | 37 |
| Pseudomonas aeruginosa AUS217 | clinical isolate | 2018 | PRJNA325248 | IPC241_RS06755 | 94.9 | 37 |
| Pseudomonas aeruginosa CN573=PSE143 | clinical isolate | 2018 | PRJNA325248 | IPC705_RS12175 | 94.9 | 37 |
| Pseudomonas aeruginosa LiA133_2003 | clinical isolate | 2018 | PRJNA325248 | IPC776_RS14055 | 95.0 | 36 |
| Pseudomonas aeruginosa 856 | clinical isolate-Veterinary | 2018 | PRJNA325248 | IPC97_RS19325 | 95.0 | 36 |
| Pseudomonas aeruginosa LMG2107 | environmental isolate | 2018 | PRJNA325248 | IPC877_RS16050 | 95.0 | 36 |

| | | | | | | |
|---|---|---|---|---|---|---|
| Pseudomonas aeruginosa PA-W16 | clinical isolate | 2018 | PRJNA325248 | IPC1286_RS00645 | 95.2 | 35 |
| Pseudomonas aeruginosa AUS021 | clinical isolate | 2018 | PRJNA325248 | IPC494_RS08310 | 95.3 | 34 |
| Pseudomonas aeruginosa AUS343 | clinical isolate | 2018 | PRJNA325248 | IPC274_RS12385 | 95.3 | 34 |
| Pseudomonas aeruginosa AUS344 | clinical isolate | 2018 | PRJNA325248 | IPC495_RS08790 | 95.3 | 34 |
| Pseudomonas aeruginosa AUS345 | clinical isolate | 2018 | PRJNA325248 | IPC496_RS11100 | 95.3 | 34 |
| Pseudomonas aeruginosa U0330A | clinical isolate | 2018 | PRJNA325248 | IPC940_RS12540 | 95.3 | 34 |
| Pseudomonas aeruginosa AUS702 | clinical isolate | 2018 | PRJNA325248 | IPC339_RS10510 | 95.4 | 33 |
| Pseudomonas aeruginosa JD303 | clinical isolate | 2018 | PRJNA198782 | DZ28_RS11045 | 95.4 | 33 |
| Pseudomonas aeruginosa JD303 | clinical isolate | 2018 | PRJNA198782 | IPC887_RS10365 | 95.4 | 33 |
| Pseudomonas aeruginosa PA-W10 | clinical isolate | 2018 | PRJNA325248 | IPC1284_RS15550 | 95.4 | 33 |
| Pseudomonas aeruginosa AUS077 | clinical isolate | 2018 | PRJNA325248 | IPC347_RS19660 | 95.6 | 32 |
| Pseudomonas aeruginosa AUS205 | clinical isolate | 2018 | PRJNA325248 | IPC224_RS09780 | 95.6 | 32 |
| Pseudomonas aeruginosa AUS229 | clinical isolate | 2018 | PRJNA325248 | IPC255_RS09390 | 95.6 | 32 |
| Pseudomonas aeruginosa AUS489 | clinical isolate | 2018 | PRJNA325248 | IPC438_RS26680 | 95.6 | 32 |
| Pseudomonas aeruginosa Us411 | clinical isolate | 2018 | PRJNA325248 | IPC860_RS00595 | 95.6 | 32 |
| Pseudomonas aeruginosa AUS476 | clinical isolate | 2018 | PRJNA325248 | IPC277_RS23265 | 95.8 | 30 |
| Pseudomonas aeruginosa PMM38 | clinical isolate | 2018 | PRJNA325248 | IPC888_RS23760 | 95.8 | 30 |
| Pseudomonas aeruginosa AL191 | clinical isolate | 2018 | PRJNA325248 | IPC1350_RS11015 | 96.0 | 29 |
| Pseudomonas aeruginosa AUS105 | clinical isolate | 2018 | PRJNA325248 | IPC354_RS03400 | 96.0 | 29 |
| Pseudomonas aeruginosa AUS321 | clinical isolate | 2018 | PRJNA325248 | IPC289_RS05100 | 96.0 | 30 |
| Pseudomonas aeruginosa DUN-015A | clinical isolate | 2018 | PRJNA325248 | IPC1027_RS03590 | 96.0 | 29 |
| Pseudomonas aeruginosa TuD199 | clinical isolate | 2018 | PRJNA325248 | IPC885_RS01120 | 96.0 | 30 |
| Pseudomonas aeruginosa JYH18 | environmental isolate | 2018 | PRJNA325248 | IPC1229_RS03970 | 96.0 | 29 |
| Pseudomonas aeruginosa JYH8 | environmental isolate | 2018 | PRJNA325248 | IPC1219_RS08280 | 96.0 | 29 |
| Pseudomonas aeruginosa 5BR2 | clinical isolate | 2018 | PRJNA325248 | IPC713_RS29705 | 96.9 | 23 |
| Pseudomonas aeruginosa AUS674 | clinical isolate | 2018 | PRJNA325248 | IPC462_RS04895 | 96.9 | 23 |
| Pseudomonas aeruginosa CPHL5083 | clinical isolate | 2018 | PRJNA325248 | IPC730_RS00590 | 96.9 | 23 |

| | | | | | | |
|---|---|---|---|---|---|---|
| Pseudomonas aeruginosa PA-W44 | clinical isolate | 2018 | PRJNA325248 | IPC1301_RS11810 | 96.9 | 23 |
| Pseudomonas aeruginosa CPHL6749 | clinical isolate | 2018 | PRJNA325248 | IPC731_RS04155 | 97.1 | 21 |
| Pseudomonas aeruginosa PA-W2 | clinical isolate | 2018 | PRJNA325248 | IPC1_RS04125 | 97.1 | 21 |
| Pseudomonas aeruginosa PA-W6 | clinical isolate | 2018 | PRJNA325248 | IPC2_RS16900 | 97.1 | 21 |
| Pseudomonas aeruginosa 886-1 | clinical isolate-Veterinary | 2018 | PRJNA325248 | IPC102_RS11090 | 97.1 | 21 |
| Pseudomonas aeruginosa AMT0020-1 | clinical isolate | 2018 | PRJNA325248 | IPC550_RS09680 | 97.2 | 20 |
| Pseudomonas aeruginosa AUS471 | clinical isolate | 2018 | PRJNA325248 | IPC282_RS23255 | 97.2 | 20 |
| Pseudomonas aeruginosa C5311 | clinical isolate | 2018 | PRJNA325248 | IPC700_RS07565 | 97.2 | 20 |
| Pseudomonas aeruginosa DUN-003B | clinical isolate | 2018 | PRJNA325248 | IPC995_RS09520 | 97.2 | 20 |
| Pseudomonas aeruginosa LiA145_2005 | clinical isolate | 2018 | PRJNA325248 | IPC780_RS14895 | 97.2 | 20 |
| Pseudomonas aeruginosa PA-W21 | clinical isolate | 2018 | PRJNA325248 | IPC1287_RS20655 | 97.2 | 20 |
| Pseudomonas aeruginosa PA-W34 | clinical isolate | 2018 | PRJNA325248 | IPC19_RS19185 | 97.2 | 20 |
| Pseudomonas aeruginosa X24509 | clinical isolate | 2018 | PRJNA219853 | Q005_03145 | 97.2 | 20 |
| Pseudomonas aeruginosa AUS183 | clinical isolate | 2018 | PRJNA325248 | IPC201_RS00640 | 97.3 | 19 |
| Pseudomonas aeruginosa CPHL10662 | clinical isolate | 2018 | PRJNA325248 | IPC737_RS12750 | 97.3 | 20 |
| Pseudomonas aeruginosa Tu61 | environmental isolate | 2018 | PRJNA325248 | IPC740_RS26650 | 97.9 | 16 |
| Pseudomonas aeruginosa AUS186 | clinical isolate | 2018 | PRJNA325248 | IPC205_RS06270 | 99.7 | 2 |
| Pseudomonas aeruginosa 1311 | clinical isolate | 2018 | PRJNA507097 | EIM02_RS21840 | 95.3 | 34 |
| Pseudomonas aeruginosa 1510 | clinical isolate | 2018 | PRJNA507097 | EIM01_RS26205 | 97.1 | 21 |
| Pseudomonas aeruginosa PA_112 | clinical isolate | 2018 | PRJNA800087 | EGJ81_RS00645 | 96.9 | 23 |
| Pseudomonas aeruginosa PA_180 | clinical isolate | 2018 | PRJNA800087 | EGJ65_RS03835 | 96.9 | 23 |
| Pseudomonas aeruginosa PA_185 | clinical isolate | 2018 | PRJNA800087 | EGJ59_RS00640 | 96.9 | 23 |
| Pseudomonas aeruginosa PA_178 | clinical isolate | 2018 | PRJNA800087 | EGP97_RS00645 | 96.9 | 23 |
| Pseudomonas aeruginosa PA_179 | clinical isolate | 2018 | PRJNA800087 | EGQ15_RS00645 | 96.9 | 23 |
| Pseudomonas aeruginosa R3839 | clinical isolate | 2018 | PRJNA508406 | EJ815_RS04760 | 95.2 | 35 |
| Pseudomonas aeruginosa R4244 | clinical isolate | 2018 | PRJNA508406 | EJ593_RS01340 | 95.2 | 35 |

| | | | | | | |
|---|---|---|---|---|---|---|
| Pseudomonas aeruginosa R4369 | clinical isolate | 2018 | PRJNA508406 | EJ782_RS24625 | 95.2 | 35 |
| Pseudomonas aeruginosa U1849 | clinical isolate | 2018 | PRJNA508406 | EJ609_RS16480 | 96.0 | 29 |
| Pseudomonas aeruginosa U3484 | clinical isolate | 2018 | PRJNA508406 | EJ611_RS31780 | 96.0 | 29 |
| Pseudomonas aeruginosa R2637 | clinical isolate | 2018 | PRJNA508406 | EKL37_RS32525 | 96.5 | 24 |
| Pseudomonas aeruginosa NCTC13715 | clinical isolate | 2018 | PRJEB6403 | EL342_RS29115 | 96.0 | 29 |
| Pseudomonas aeruginosa MRSN3705 | clinical isolate | 2018 | PRJNA273956 | DY956_RS04875 | 95.0 | 36 |
| Pseudomonas aeruginosa MRSN16740 | clinical isolate | 2018 | PRJNA273956 | DY994_RS04105 | 95.3 | 34 |
| Pseudomonas aeruginosa MRSN19711 | clinical isolate | 2018 | PRJNA273956 | DY977_RS01420 | 95.3 | 34 |
| Pseudomonas aeruginosa MRSN369569 | clinical isolate | 2018 | PRJNA273956 | DY957_RS17170 | 95.3 | 34 |
| Pseudomonas aeruginosa MRSN6241 | clinical isolate | 2018 | PRJNA273956 | DY940_RS06790 | 95.3 | 34 |
| Pseudomonas aeruginosa MRSN8141 | clinical isolate | 2018 | PRJNA273956 | DY932_RS10930 | 95.3 | 34 |
| Pseudomonas aeruginosa MRSN5508 | clinical isolate | 2018 | PRJNA273956 | DY946_RS05460 | 95.4 | 33 |
| Pseudomonas aeruginosa MRSN13488 | clinical isolate | 2018 | PRJNA273956 | DZA10_RS03825 | 95.6 | 32 |
| Pseudomonas aeruginosa MRSN1613 | clinical isolate | 2018 | PRJNA273956 | DY999_RS02670 | 95.6 | 32 |
| Pseudomonas aeruginosa MRSN20176 | clinical isolate | 2018 | PRJNA273956 | DZA25_RS10070 | 96.0 | 30 |
| Pseudomonas aeruginosa MRSN8912 | clinical isolate | 2018 | PRJNA273956 | DY931_RS08655 | 96.0 | 29 |
| Pseudomonas aeruginosa MRSN16344 | clinical isolate | 2018 | PRJNA273956 | DY997_RS03500 | 96.1 | 28 |
| Pseudomonas aeruginosa MRSN11278 | clinical isolate | 2018 | PRJNA273956 | DZA23_RS01915 | 96.6 | 25 |
| Pseudomonas aeruginosa MRSN11285 | clinical isolate | 2018 | PRJNA273956 | DZA21_RS14345 | 96.6 | 25 |
| Pseudomonas aeruginosa MRSN11538 | clinical isolate | 2018 | PRJNA273956 | DZA18_RS22945 | 96.9 | 23 |
| Pseudomonas aeruginosa MRSN1356 | clinical isolate | 2018 | PRJNA273956 | DZA09_RS23670 | 97.1 | 21 |
| Pseudomonas aeruginosa MRSN18562 | clinical isolate | 2018 | PRJNA273956 | DY987_RS29640 | 97.1 | 21 |
| Pseudomonas aeruginosa MRSN1380 | clinical isolate | 2018 | PRJNA273956 | DZA08_RS09640 | 97.2 | 20 |
| Pseudomonas aeruginosa MRSN18970 | clinical isolate | 2018 | PRJNA273956 | DZA24_RS27465 | 97.2 | 17 |
| Pseudomonas aeruginosa MRSN6739 | clinical isolate | 2018 | PRJNA273956 | DY937_RS12825 | 97.2 | 20 |
| Pseudomonas aeruginosa MRSN8139 | clinical isolate | 2018 | PRJNA273956 | DY933_RS24860 | 97.2 | 20 |
| Pseudomonas aeruginosa MRSN8914 | clinical isolate | 2018 | PRJNA273956 | DY930_RS05050 | 97.2 | 20 |

| | | | | | | |
|---|---|---|---|---|---|---|
| Pseudomonas aeruginosa MRSN436311 | clinical isolate | 2018 | PRJNA273956 | DY950_RS08710 | 98.9 | 8 |
| Pseudomonas aeruginosa JYH17 | environmental isolate | 2018 | PRJNA325248 | IPC1228_RS02240 | 89.4 | 76 |
| Pseudomonas aeruginosa AUS464 | clinical isolate | 2018 | PRJNA325248 | IPC432_RS05025 | 95.2 | 35 |
| Pseudomonas aeruginosa AUS176 | clinical isolate | 2018 | PRJNA325248 | IPC413_RS00590 | 95.3 | 34 |
| Pseudomonas aeruginosa AUS527 | clinical isolate | 2018 | PRJNA325248 | IPC324_RS06460 | 95.3 | 34 |
| Pseudomonas aeruginosa T3354 | clinical isolate | 2018 | PRJNA325248 | IPC1135_RS09810 | 95.3 | 34 |
| Pseudomonas aeruginosa AUS158 | clinical isolate | 2018 | PRJNA325248 | IPC397_RS07340 | 95.4 | 33 |
| Pseudomonas aeruginosa AUS526 | clinical isolate | 2018 | PRJNA325248 | IPC450_RS25865 | 95.4 | 33 |
| Pseudomonas aeruginosa HCF591 | clinical isolate | 2018 | PRJNA325248 | IPC1421_RS13865 | 95.4 | 33 |
| Pseudomonas aeruginosa MCF922 | clinical isolate | 2018 | PRJEB45250 | IPC1362_RS00600 | 95.4 | 33 |
| Pseudomonas aeruginosa AUS110 | clinical isolate | 2018 | PRJNA325248 | IPC357_RS09015 | 95.6 | 32 |
| Pseudomonas aeruginosa AUS175 | clinical isolate | 2018 | PRJNA325248 | IPC412_RS07955 | 95.6 | 32 |
| Pseudomonas aeruginosa AUS265 | clinical isolate | 2018 | PRJNA325248 | IPC325_RS07220 | 95.6 | 32 |
| Pseudomonas aeruginosa AUS500 | clinical isolate | 2018 | PRJNA325248 | IPC443_RS02280 | 95.6 | 32 |
| Pseudomonas aeruginosa AUS510 | clinical isolate | 2018 | PRJNA325248 | IPC447_RS02675 | 95.6 | 32 |
| Pseudomonas aeruginosa HCF410 | clinical isolate | 2018 | PRJNA325248 | IPC1417_RS03890 | 95.6 | 32 |
| Pseudomonas aeruginosa AUS122 | clinical isolate | 2018 | PRJNA325248 | IPC367_RS12110 | 96.0 | 29 |
| Pseudomonas aeruginosa AUS449 | clinical isolate | 2018 | PRJNA325248 | IPC451_RS14035 | 96.0 | 29 |
| Pseudomonas aeruginosa Zw64 | clinical isolate | 2018 | PRJNA325248 | IPC1384_RS08900 | 96.0 | 29 |
| Pseudomonas aeruginosa JYH29 | environmental isolate | 2018 | PRJNA325248 | IPC1240_RS12295 | 96.0 | 29 |
| Pseudomonas aeruginosa T4242 | clinical isolate | 2018 | PRJNA325248 | IPC1134_RS09295 | 96.1 | 28 |
| Pseudomonas aeruginosa AUS227 | clinical isolate | 2018 | PRJNA325248 | IPC253_RS17740 | 96.2 | 27 |
| Pseudomonas aeruginosa AUS221 | clinical isolate | 2018 | PRJNA325248 | IPC245_RS06575 | 96.5 | 26 |
| Pseudomonas aeruginosa Zw73_2 | clinical isolate | 2018 | PRJNA325248 | IPC1437_RS21450 | 96.8 | 24 |
| Pseudomonas aeruginosa W15Aug23 | environmental isolate | 2018 | PRJNA325248 | IPC825_RS00540 | 96.9 | 23 |
| Pseudomonas aeruginosa BJ4 | clinical isolate | 2018 | PRJNA325248 | IPC1423_RS09560 | 97.1 | 21 |
| Pseudomonas aeruginosa H2 | clinical isolate | 2018 | PRJNA325248 | IPC1375_RS06415 | 97.1 | 21 |

| | | | | | | |
|---|---|---|---|---|---|---|
| Pseudomonas aeruginosa MCF134 | clinical isolate | 2018 | PRJEB45250 | IPC1364_RS11820 | 97.1 | 21 |
| Pseudomonas aeruginosa AUS153 | clinical isolate | 2018 | PRJNA325248 | IPC392_RS24980 | 97.2 | 20 |
| Pseudomonas aeruginosa AUS155 | clinical isolate | 2018 | PRJNA325248 | IPC394_RS27140 | 97.2 | 20 |
| Pseudomonas aeruginosa JYH25 | environmental isolate | 2018 | PRJNA325248 | IPC1236_RS04815 | 97.2 | 20 |
| Pseudomonas aeruginosa PT12 | environmental isolate | 2018 | PRJNA325248 | IPC1387_RS27495 | 97.2 | 20 |
| Pseudomonas aeruginosa AUS512 | clinical isolate | 2018 | PRJNA325248 | IPC449_RS25140 | 99.7 | 2 |
| Pseudomonas aeruginosa FM4 | clinical isolate | 2018 | PRJNA325248 | IPC1429_RS09475 | 99.7 | 2 |
| Pseudomonas aeruginosa JYH11 | environmental isolate | 2018 | PRJNA325248 | IPC1222_RS15940 | 99.9 | 1 |
| Pseudomonas aeruginosa LW | clinical isolate | 2019 | PRJNA391028 | CEK59_RS14795 | 96.6 | 25 |
| Pseudomonas aeruginosa Pae28 | clinical isolate | 2019 | PRJNA514718 | EQH73_RS07150 | 95.4 | 33 |
| Pseudomonas aeruginosa Pae39 | clinical isolate | 2019 | PRJNA514718 | EQH75_RS11795 | 95.4 | 33 |
| Pseudomonas aeruginosa Pae83 | clinical isolate | 2019 | PRJNA514718 | EQH80_RS08780 | 95.4 | 33 |
| Pseudomonas aeruginosa Pae21 | clinical isolate | 2019 | PRJNA514718 | EQH70_RS09640 | 95.6 | 32 |
| Pseudomonas aeruginosa Pae81 | clinical isolate | 2019 | PRJNA514718 | EQH79_RS22780 | 95.8 | 30 |
| Pseudomonas aeruginosa Pae66 | clinical isolate | 2019 | PRJNA514718 | EQH71_RS01370 | 96.2 | 27 |
| Pseudomonas aeruginosa M53 | environmental isolate | 2019 | PRJNA517176 | EWP69_RS24705 | 96.5 | 26 |
| Pseudomonas aeruginosa M54 | environmental isolate | 2019 | PRJNA517176 | EWI34_RS25075 | 96.5 | 26 |
| Pseudomonas aeruginosa M55 | environmental isolate | 2019 | PRJNA517176 | EWI54_RS22595 | 96.5 | 26 |
| Pseudomonas aeruginosa M56 | environmental isolate | 2019 | PRJNA517176 | EWP46_RS25470 | 96.5 | 26 |
| Pseudomonas aeruginosa M57 | environmental isolate | 2019 | PRJNA517176 | EWI29_RS23250 | 96.5 | 26 |
| Pseudomonas aeruginosa M58 | environmental isolate | 2019 | PRJNA517176 | EWI28_RS28915 | 96.5 | 26 |
| Pseudomonas aeruginosa M59 | environmental isolate | 2019 | PRJNA517176 | EWI33_RS12035 | 96.5 | 26 |
| Pseudomonas aeruginosa M60 | environmental isolate | 2019 | PRJNA517176 | EWM61_RS11105 | 96.5 | 26 |
| Pseudomonas aeruginosa M61 | environmental isolate | 2019 | PRJNA517176 | EWP22_RS23455 | 96.5 | 26 |
| Pseudomonas aeruginosa M62 | environmental isolate | 2019 | PRJNA517176 | EWI35_RS22285 | 96.5 | 26 |

| | | | | | | |
|---|---|---|---|---|---|---|
| Pseudomonas aeruginosa 13 | clinical isolate | 2019 | PRJNA325248、PRJEB5438、PRJNA514442 | EQ530_RS06255 | 95.3 | 34 |
| Pseudomonas aeruginosa isolate 17 | clinical isolate | 2019 | PRJEB5438 | BUG75_RS21250 | 95.4 | 33 |
| Pseudomonas aeruginosa isolate 18 | clinical isolate | 2019 | PRJEB5438 | BUG87_RS09045 | 95.4 | 33 |
| Pseudomonas aeruginosa isolate 19 | clinical isolate | 2019 | PRJEB5438 | BUH20_RS28400 | 95.4 | 33 |
| Pseudomonas aeruginosa isolate 20 | clinical isolate | 2019 | PRJEB5438 | BUH17_RS04585 | 95.4 | 33 |
| Pseudomonas aeruginosa 95 | clinical isolate | 2019 | PRJEB5438、PRJNA514442 | EQ608_RS07845 | 95.6 | 32 |
| Pseudomonas aeruginosa isolate 76 | clinical isolate | 2019 | PRJEB5438 | BUR95_RS22755 | 96.6 | 25 |
| Pseudomonas aeruginosa isolate 77 | clinical isolate | 2019 | PRJEB5438 | BUR30_RS12855 | 96.6 | 25 |
| Pseudomonas aeruginosa isolate 78 | clinical isolate | 2019 | PRJEB5438 | BUQ80_RS26325 | 96.6 | 25 |
| Pseudomonas aeruginosa isolate 79 | clinical isolate | 2019 | PRJEB5438 | BUR21_RS24145 | 96.6 | 25 |
| Pseudomonas aeruginosa isolate 80 | clinical isolate | 2019 | PRJEB5438 | BUR22_RS23290 | 96.6 | 25 |
| Pseudomonas aeruginosa isolate 82 | clinical isolate | 2019 | PRJEB5438 | BUQ86_RS22155 | 96.6 | 25 |
| Pseudomonas aeruginosa isolate 83 | clinical isolate | 2019 | PRJEB5438 | BUR00_RS18615 | 96.6 | 25 |
| Pseudomonas aeruginosa isolate 84 | clinical isolate | 2019 | PRJEB5438 | BUS00_RS25635 | 96.6 | 25 |
| Pseudomonas aeruginosa isolate 86 | clinical isolate | 2019 | PRJEB5438 | BUR36_RS24770 | 96.6 | 25 |
| Pseudomonas aeruginosa isolate 87 | clinical isolate | 2019 | PRJEB5438 | BUS17_RS28410 | 96.6 | 25 |
| Pseudomonas aeruginosa isolate 88 | clinical isolate | 2019 | PRJEB5438 | BUR23_RS13115 | 96.6 | 25 |
| Pseudomonas aeruginosa isolate 90 | clinical isolate | 2019 | PRJEB5438 | BUR43_RS23625 | 96.6 | 25 |
| Pseudomonas aeruginosa 133 | clinical isolate | 2019 | PRJNA514442 | EQ642_RS06300 | 97.1 | 21 |
| Pseudomonas aeruginosa isolate 124 | clinical isolate | 2019 | PRJEB5438 | BUF36_RS24345 | 97.1 | 21 |
| Pseudomonas aeruginosa isolate 126 | clinical isolate | 2019 | PRJEB5438 | BUG13_RS10245 | 97.1 | 21 |
| Pseudomonas aeruginosa isolate 127 | clinical isolate | 2019 | PRJEB5438 | BUH14_RS00545 | 97.1 | 21 |
| Pseudomonas aeruginosa isolate 128 | clinical isolate | 2019 | PRJEB5438 | BUF99_RS22710 | 97.1 | 21 |
| Pseudomonas aeruginosa isolate 129 | clinical isolate | 2019 | PRJEB5438 | BUG45_RS23225 | 97.1 | 21 |

| | | | | | | |
|---|---|---|---|---|---|---|
| Pseudomonas aeruginosa isolate 131 | clinical isolate | 2019 | PRJEB5438 | BUG39_RS25610 | 97.1 | 21 |
| Pseudomonas aeruginosa isolate 132 | clinical isolate | 2019 | PRJEB5438 | BUF77_RS25305 | 97.1 | 21 |
| Pseudomonas aeruginosa 10 | clinical isolate | 2019 | PRJNA325248、PRJEB5438、PRJNA514442 | EQ527_RS20820 | 97.2 | 20 |
| Pseudomonas aeruginosa 17 | clinical isolate | 2019 | PRJNA325248、PRJEB5438、PRJNA514442 | EQ533_RS15545 | 97.2 | 20 |
| Pseudomonas aeruginosa 26 | clinical isolate | 2019 | PRJNA342804、PRJNA514442 | EQ541_RS01685 | 97.2 | 20 |
| Pseudomonas aeruginosa PAC76A | clinical isolate | 2019 | PRJNA325248 | IPC177_RS28430 | 95.3 | 34 |
| Pseudomonas aeruginosa 4 | clinical isolate | 2019 | PRJNA325248、PRJNA514442 | IPC1586_RS00595 | 95.4 | 33 |
| Pseudomonas aeruginosa isolate 16 | clinical isolate | 2019 | PRJEB5438 | BUG83_RS23500 | 95.4 | 33 |
| Pseudomonas aeruginosa 18G | environmental isolate | 2019 | PRJNA325248 | IPC574_RS02675 | 95.4 | 33 |
| Pseudomonas aeruginosa 19R | environmental isolate | 2019 | PRJNA325248 | IPC577_RS02680 | 95.4 | 33 |
| Pseudomonas aeruginosa 19SJV | environmental isolate | 2019 | PRJNA325248 | IPC576_RS01840 | 95.4 | 33 |
| Pseudomonas aeruginosa 19SV | environmental isolate | 2019 | PRJNA325248 | IPC578_RS02675 | 95.4 | 33 |
| Pseudomonas aeruginosa 32SB | environmental isolate | 2019 | PRJNA325248 | IPC580_RS02675 | 95.4 | 33 |
| Pseudomonas aeruginosa 32SP | environmental isolate | 2019 | PRJNA325248 | IPC579_RS03110 | 95.4 | 33 |
| Pseudomonas aeruginosa 34JS | environmental isolate | 2019 | PRJNA325248 | IPC582_RS02675 | 95.4 | 33 |
| Pseudomonas aeruginosa 57RV | environmental isolate | 2019 | PRJNA325248 | IPC586_RS02680 | 95.4 | 33 |
| Pseudomonas aeruginosa 57SJ | environmental isolate | 2019 | PRJNA325248 | IPC584_RS03075 | 95.4 | 33 |
| Pseudomonas aeruginosa 78RV | environmental isolate | 2019 | PRJNA325248 | IPC589_RS02680 | 95.4 | 33 |
| Pseudomonas aeruginosa PAC5A | clinical isolate | 2019 | PRJNA325248 | IPC112_RS09155 | 95.8 | 30 |
| Pseudomonas aeruginosa PAC46A | clinical isolate | 2019 | PRJNA325248 | IPC114_RS08985 | 96.0 | 29 |
| Pseudomonas aeruginosa PAC5B | clinical isolate | 2019 | PRJNA325248 | IPC113_RS18860 | 96.0 | 29 |

| | | | | | | |
|---|---|---|---|---|---|---|
| Pseudomonas aeruginosa PAC61A | clinical isolate | 2019 | PRJNA325248 | IPC115_RS19315 | 96.0 | 29 |
| Pseudomonas aeruginosa 145S200511BSL_PA2 | clinical isolate | 2019 | PRJNA325248 | IPC40_RS09865 | 96.1 | 28 |
| Pseudomonas aeruginosa 5023272159 | clinical isolate | 2019 | PRJNA325248 | IPC1514_RS10465 | 96.9 | 19 |
| Pseudomonas aeruginosa 5995 | clinical isolate | 2019 | PRJNA325248 | IPC1318_RS06035 | 97.1 | 21 |
| Pseudomonas aeruginosa 5996 | clinical isolate | 2019 | PRJNA325248 | IPC1319_RS24690 | 97.1 | 21 |
| Pseudomonas aeruginosa 6004 | clinical isolate | 2019 | PRJNA325248 | IPC1330_RS05230 | 97.1 | 21 |
| Pseudomonas aeruginosa 6095 | clinical isolate | 2019 | PRJNA325248 | IPC1342_RS18940 | 97.1 | 21 |
| Pseudomonas aeruginosa PAC31B | clinical isolate | 2019 | PRJNA325248 | IPC163_RS10045 | 97.1 | 21 |
| Pseudomonas aeruginosa PAC42A | clinical isolate | 2019 | PRJNA325248 | IPC164_RS00590 | 97.1 | 21 |
| Pseudomonas aeruginosa PAC70A | clinical isolate | 2019 | PRJNA325248 | IPC165_RS10050 | 97.1 | 21 |
| Pseudomonas aeruginosa PAC70B | clinical isolate | 2019 | PRJNA325248 | IPC166_RS11340 | 97.1 | 21 |
| Pseudomonas aeruginosa PAC80A | clinical isolate | 2019 | PRJNA325248 | IPC167_RS09645 | 97.1 | 21 |
| Pseudomonas aeruginosa PAC98B | clinical isolate | 2019 | PRJNA325248 | IPC169_RS10045 | 97.1 | 21 |
| Pseudomonas aeruginosa PAC98C | clinical isolate | 2019 | PRJNA325248 | IPC170_RS00590 | 97.1 | 21 |
| Pseudomonas aeruginosa PAC98D | clinical isolate | 2019 | PRJNA325248 | IPC171_RS00590 | 97.1 | 21 |
| Pseudomonas aeruginosa 11 | clinical isolate | 2019 | PRJEB5438 | IPC1593_RS12980 | 97.2 | 20 |
| Pseudomonas aeruginosa 313s141011BSL_PA1 | clinical isolate | 2019 | PRJNA325248 | IPC70_RS13275 | 97.2 | 20 |
| Pseudomonas aeruginosa 6001 | clinical isolate | 2019 | PRJNA325248 | IPC1328_RS08780 | 97.2 | 20 |
| Pseudomonas aeruginosa 6092.2 | clinical isolate | 2019 | PRJNA325248 | IPC1339_RS09385 | 97.2 | 20 |
| Pseudomonas aeruginosa 6092.3 | clinical isolate | 2019 | PRJNA325248 | IPC1340_RS03965 | 97.2 | 20 |
| Pseudomonas aeruginosa 19SJO | environmental isolate | 2019 | PRJNA325248 | IPC575_RS02685 | 95.4 | 33 |
| Pseudomonas aeruginosa PAC31A | clinical isolate | 2019 | PRJNA325248 | IPC162_RS02625 | 97.1 | 21 |
| Pseudomonas aeruginosa MMK2018 | clinical isolate | 2019 | PRJNA530358 | E5D53_RS04715 | 95.3 | 34 |
| Pseudomonas aeruginosa isolate 91 | clinical isolate | 2019 | PRJEB5438 | BUR13_RS28920 | 96.6 | 25 |
| Pseudomonas aeruginosa isolate 81 | clinical isolate | 2019 | PRJEB5438 | BUR50_RS25705 | 96.6 | 25 |

| | | | | | | |
|---|---|---|---|---|---|---|
| Pseudomonas aeruginosa isolate 89 | clinical isolate | 2019 | PRJEB5438 | BUR25_RS17895 | 96.6 | 25 |
| Pseudomonas aeruginosa isolate 123 | clinical isolate | 2019 | PRJEB5438 | BUG98_RS20740 | 97.1 | 21 |
| Pseudomonas aeruginosa isolate 125 | clinical isolate | 2019 | PRJEB5438 | BUF98_RS23745 | 97.1 | 21 |
| Pseudomonas aeruginosa NCTC11839 | clinical isolate | 2019 | PRJEB6403 | FQU16_RS31100 | 97.1 | 21 |
| Pseudomonas aeruginosa EPIC E190 | clinical isolate | 2019 | PRJNA471888 | DMY50_RS14045 | 97.1 | 21 |
| Pseudomonas aeruginosa EPIC E54 | clinical isolate | 2019 | PRJNA471888 | DMY46_RS06670 | 97.2 | 20 |
| Pseudomonas aeruginosa GO74 | clinical isolate | 2019 | PRJNA552551 | FNL70_RS02340 | 95.6 | 32 |
| Pseudomonas aeruginosa UY1PSABAL | clinical isolate | 2019 | PRJNA554269 | FNV45_RS12930 | 96.0 | 30 |
| Pseudomonas aeruginosa 60503 | clinical isolate | 2019 | PRJNA342804 | FOY97_RS20460 | 96.0 | 29 |
| Pseudomonas aeruginosa SCAID WND1-2019 | clinical isolate | 2019 | PRJNA554979 | FPJ23_RS11420 | 97.0 | 18 |
| Pseudomonas aeruginosa C79 | clinical isolate | 2019 | PRJNA544424 | FF962_RS02610 | 95.7 | 32 |
| Pseudomonas aeruginosa 6487 | clinical isolate | 2019 | PRJNA558357 | FSB74_RS18450 | 95.3 | 34 |
| Pseudomonas aeruginosa 6354 | clinical isolate | 2019 | PRJNA558357 | FSC46_RS20300 | 96.0 | 29 |
| Pseudomonas aeruginosa isolate 335 | clinical isolate | 2019 | PRJEB5438 | BUI80_RS21570 | 97.1 | 21 |
| Pseudomonas aeruginosa L30 | clinical isolate | 2019 | PRJNA564419 | F3H09_RS01825 | 95.3 | 34 |
| Pseudomonas aeruginosa L14 | clinical isolate | 2019 | PRJNA564419 | F3G61_RS14570 | 95.4 | 33 |
| Pseudomonas aeruginosa L3 | clinical isolate | 2019 | PRJNA564419 | F3G62_RS03140 | 95.4 | 33 |
| Pseudomonas aeruginosa L7 | clinical isolate | 2019 | PRJNA564419 | F3G89_RS06140 | 96.0 | 30 |
| Pseudomonas aeruginosa L35 | clinical isolate | 2019 | PRJNA564419 | F3G51_RS14970 | 96.6 | 24 |
| Pseudomonas aeruginosa L36 | clinical isolate | 2019 | PRJNA564419 | F3G54_RS18835 | 96.6 | 24 |
| Pseudomonas aeruginosa P38 | clinical isolate | 2019 | PRJNA564463 | F3G93_RS11970 | 96.6 | 24 |
| Pseudomonas aeruginosa MUC-P4 | clinical isolate | 2019 | PRJNA575806 | GAB06_RS03675 | 97.2 | 20 |
| Pseudomonas aeruginosa GO79 | clinical isolate | 2019 | PRJNA552551 | F8139_RS17070 | 97.2 | 20 |
| Pseudomonas aeruginosa GO68 | clinical isolate | 2019 | PRJNA552551 | F8136_RS03085 | 97.1 | 21 |
| Pseudomonas aeruginosa PA45 | clinical isolate | 2019 | PRJNA189678 | H734_02392 | 95.3 | 34 |
| Pseudomonas aeruginosa PA120 | clinical isolate | 2019 | PRJNA556255 | FPQ12_RS03950 | 96.6 | 25 |

| | | | | | | |
|---|---|---|---|---|---|---|
| Pseudomonas aeruginosa PA155 | clinical isolate | 2019 | PRJNA550403 | GFH40_RS03985 | 97.1 | 21 |
| Pseudomonas aeruginosa PA141 | clinical isolate | 2019 | PRJNA550403 | GFH38_RS06230 | 97.2 | 20 |
| Pseudomonas aeruginosa 2600 | clinical isolate | 2019 | PRJNA562202 | FY178_RS00755 | 96.9 | 23 |
| Pseudomonas aeruginosa ST773 | clinical isolate | 2019 | PRJNA555326 | FOZ66_RS20245 | 96.0 | 29 |
| Pseudomonas aeruginosa PA206 | clinical isolate | 2019 | PRJNA846971 | GNQ20_RS07650 | 89.7 | 74 |
| Pseudomonas aeruginosa PA123 | clinical isolate | 2019 | PRJNA590804 | GNQ09_RS03060 | 95.6 | 32 |
| Pseudomonas aeruginosa PA193 | clinical isolate | 2019 | PRJNA590804 | GNQ25_RS16070 | 95.6 | 32 |
| Pseudomonas aeruginosa PA202 | clinical isolate | 2019 | PRJNA590804 | GNQ40_RS02605 | 96.0 | 30 |
| Pseudomonas aeruginosa PA220 | clinical isolate | 2019 | PRJNA590804 | GNQ36_RS02615 | 96.0 | 30 |
| Pseudomonas aeruginosa PA221 | clinical isolate | 2019 | PRJNA590804 | GNQ48_RS02605 | 96.0 | 30 |
| Pseudomonas aeruginosa PA218 | clinical isolate | 2019 | PRJNA590804 | GNQ28_RS09315 | 96.1 | 28 |
| Pseudomonas aeruginosa PA126 | clinical isolate | 2019 | PRJNA590804 | GNQ12_RS24135 | 97.1 | 21 |
| Pseudomonas aeruginosa MZ4A | clinical isolate | 2019 | PRJNA589626 | GJ673_RS19420 | 96.1 | 28 |
| Pseudomonas aeruginosa P8 | clinical isolate | 2019 | PRJNA562233 | FZE41_RS18605 | 95.6 | 32 |
| Pseudomonas aeruginosa PA-50010278 | clinical isolate | 2019 | PRJNA595618 | GQF67_RS29810 | 96.0 | 29 |
| Pseudomonas aeruginosa Pb9 | clinical isolate | 2020 | PRJNA595937 | PAPB9_RS21195 | 95.6 | 32 |
| Pseudomonas aeruginosa VNMU089 | clinical isolate | 2020 | PRJNA598234 | GUL14_RS14940 | 95.6 | 32 |
| Pseudomonas aeruginosa BCW_7428 | clinical isolate-Veterinary | 2020 | PRJNA203445 | EON08_RS15445 | 95.2 | 35 |
| Pseudomonas aeruginosa BCW_7430 | clinical isolate-Veterinary | 2020 | PRJNA203445 | EON10_RS28010 | 96.5 | 26 |
| Pseudomonas aeruginosa BCW_7427 | clinical isolate-Veterinary | 2020 | PRJNA203445 | EON07_RS18575 | 96.5 | 26 |
| Pseudomonas aeruginosa NICED-PA-01 | clinical isolate | 2020 | PRJNA602579 | GWP79_RS08320 | 97.3 | 20 |
| Pseudomonas aeruginosa PABL046 | clinical isolate | 2020 | PRJNA485889 | DZ925_RS17145 | 95.3 | 34 |
| Pseudomonas aeruginosa PABL096 | clinical isolate | 2020 | PRJNA485889 | DZ973_RS23490 | 95.6 | 32 |
| Pseudomonas aeruginosa PABL043 | clinical isolate | 2020 | PRJNA485889 | DZ922_RS24140 | 96.6 | 24 |

| | | | | | | |
|---|---|---|---|---|---|---|
| Pseudomonas aeruginosa PABL018 | clinical isolate | 2020 | PRJNA485889 | DZ900_RS10850 | 96.9 | 23 |
| Pseudomonas aeruginosa PABL007 | clinical isolate | 2020 | PRJNA485889 | DZ890_RS14520 | 97.1 | 21 |
| Pseudomonas aeruginosa PABL028 | clinical isolate | 2020 | PRJNA485889 | DZ909_RS16125 | 97.1 | 21 |
| Pseudomonas aeruginosa PABL030 | clinical isolate | 2020 | PRJNA485889 | DZ911_RS19045 | 97.1 | 21 |
| Pseudomonas aeruginosa PABL076 | clinical isolate | 2020 | PRJNA485889 | DZ954_RS25155 | 97.1 | 21 |
| Pseudomonas aeruginosa PABL003 | clinical isolate | 2020 | PRJNA485889 | DZ887_RS26700 | 97.2 | 20 |
| Pseudomonas aeruginosa PABL013 | clinical isolate | 2020 | PRJNA485889 | DZ895_RS22885 | 97.2 | 20 |
| Pseudomonas aeruginosa PABL061 | clinical isolate | 2020 | PRJNA485889 | DZ939_RS22600 | 97.2 | 20 |
| Pseudomonas aeruginosa PABL102 | clinical isolate | 2020 | PRJNA485889 | DZ978_RS16220 | 95.6 | 32 |
| Pseudomonas aeruginosa PS75 | clinical isolate | 2022 | PRJNA227544 | V562_03006 | 97.2 | 20 |
| Pseudomonas aeruginosa isolate G | clinical isolate | 2022 | PRJNA719713 | BUR86_RS30810 | 96.6 | 25 |
| Pseudomonas sp. YS-1p | environmental isolate | | PRJNA257546 | JL38_RS12870 | 94.9 | 37 |
| Pseudomonas aeruginosa isolate 418 | clinical isolate | | PRJEB5438 | BUJ71_RS13495 | 95.2 | 35 |
| Pseudomonas aeruginosa isolate 422 | clinical isolate | | PRJEB5438 | BUJ86_RS15820 | 95.2 | 35 |
| Pseudomonas aeruginosa isolate 423.1 | clinical isolate | | PRJEB5438 | BUK12_RS23965 | 95.2 | 35 |
| Pseudomonas aeruginosa isolate 424 | clinical isolate | | PRJEB5438 | C4T26_RS25060 | 95.2 | 35 |
| Pseudomonas aeruginosa isolate 426 | clinical isolate | | PRJEB5438 | BUQ84_RS14775 | 95.2 | 35 |
| Pseudomonas aeruginosa isolate 428 | clinical isolate | | PRJEB5438 | BUR15_RS15145 | 95.2 | 35 |
| Pseudomonas aeruginosa isolate 430 | clinical isolate | | PRJEB5438 | BUQ75_RS20440 | 95.2 | 35 |
| Pseudomonas aeruginosa isolate 431 | clinical isolate | | PRJEB5438 | BUR31_RS23915 | 95.2 | 35 |
| Pseudomonas aeruginosa isolate 434 | clinical isolate | | PRJEB5438 | BUR46_RS13260 | 95.2 | 35 |
| Pseudomonas aeruginosa isolate 437 | clinical isolate | | PRJEB5438 | BUQ83_RS26970 | 95.2 | 35 |
| Pseudomonas aeruginosa isolate 437.1 | clinical isolate | | PRJEB5438 | BUR26_RS26535 | 95.2 | 35 |
| Pseudomonas aeruginosa isolate 439 | clinical isolate | | PRJEB5438 | BUR10_RS18175 | 95.2 | 35 |
| Pseudomonas aeruginosa 192S190811BSL_PA2 | clinical isolate | | PRJNA325248 | IPC44_RS03820 | 95.4 | 33 |
| Pseudomonas aeruginosa isolate LRJ05 | clinical isolate | | PRJEB5438 | BUS47_RS26175 | 95.4 | 33 |

| | | | | | |
|---|---|---|---|---|---|
| Pseudomonas aeruginosa XDR-PA / assembly CAT08-025 | clinical isolate | PRJEB31047 | E4Z46_RS03935 | 95.4 | 33 |
| Pseudomonas sp HMSC076A11 | clinical isolate | PRJNA296326 | HMPREF2716_RS18335 | 95.4 | 33 |
| Pseudomonas sp HMSC076A12 | clinical isolate | PRJNA296326 | HMPREF2840_RS08910 | 95.4 | 33 |
| Pseudomonas aeruginosa 6D92 | clinical isolate | PRJNA325248 | IPC1260_RS00185 | 95.6 | 32 |
| Pseudomonas sp HMSC060G02 | clinical isolate | PRJNA296326 | HMPREF2719_RS24195 | 95.6 | 32 |
| Pseudomonas aeruginosa isolate RW204 | environmental isolate | PRJEB8749 | AQT53_RS12370 | 95.6 | 32 |
| Pseudomonas aeruginosa XDR-PA / assembly CAT01-001 | clinical isolate | PRJEB31047 | E4Z49_RS20850 | 96.0 | 29 |
| Pseudomonas aeruginosa XDR-PA / assembly MAD04-016 | clinical isolate | PRJEB31047 | E4018_RS23060 | 96.0 | 29 |
| Pseudomonas aeruginosa isolate 10% 5 | environmental isolate | PRJEB8749 | AQT03_RS12375 | 96.0 | 30 |
| Pseudomonas sp. 2VD | environmental isolate | PRJNA414404 | FFS27_RS17895 | 96.0 | 30 |
| Pseudomonas aeruginosa A1 | clinical isolate | PRJNA818324 | IPC1122_RS25405 | 96.1 | 28 |
| Pseudomonas aeruginosa isolate 187 | clinical isolate | PRJEB5438 | BUH22_RS22630 | 96.1 | 28 |
| Pseudomonas aeruginosa XDR-PA / assembly AND04-037 | clinical isolate | PRJEB31047 | E4Y84_RS23555 | 96.5 | 26 |
| Pseudomonas aeruginosa isolate 81.1 | clinical isolate | PRJEB5438 | BUR89_RS27395 | 96.6 | 25 |
| Pseudomonas aeruginosa isolate 82.1 | clinical isolate | PRJEB5438 | BUR59_RS23460 | 96.6 | 25 |
| Pseudomonas aeruginosa isolate A | clinical isolate | PRJNA719713 | BUS20_RS30090 | 96.6 | 25 |
| Pseudomonas aeruginosa isolate B | clinical isolate | PRJNA719713 | BUR33_RS28720 | 96.6 | 25 |
| Pseudomonas aeruginosa isolate C | clinical isolate | PRJNA719713 | BUR96_RS28445 | 96.6 | 25 |
| Pseudomonas aeruginosa isolate D | clinical isolate | PRJNA719713 | BUS58_RS21495 | 96.6 | 25 |
| Pseudomonas aeruginosa isolate E | clinical isolate | PRJNA719713 | BUR55_RS31375 | 96.6 | 25 |
| Pseudomonas aeruginosa isolate F | clinical isolate | PRJNA719713 | BUS15_RS30475 | 96.6 | 25 |
| Pseudomonas aeruginosa isolate H | clinical isolate | PRJNA719713 | BUR16_RS29135 | 96.6 | 25 |
| Pseudomonas aeruginosa isolate Ii | clinical isolate | PRJEB5438 | BUR40_RS29365 | 96.6 | 25 |

| | | | | | |
|---|---|---|---|---|---|
| Pseudomonas aeruginosa isolate J | clinical isolate | PRJEB5438 | BUS19_RS29120 | 96.6 | 25 |
| Pseudomonas aeruginosa isolate K | clinical isolate | PRJEB5438 | BUR56_RS02605 | 96.6 | 25 |
| Pseudomonas aeruginosa isolate L | clinical isolate | PRJEB5438 | BUR90_RS28860 | 96.6 | 25 |
| Pseudomonas aeruginosa isolate LRJ14 | clinical isolate | PRJEB5438 | BUS32_RS16675 | 96.6 | 25 |
| Pseudomonas aeruginosa isolate LRJ15 | clinical isolate | PRJEB5438 | BUR88_RS21970 | 96.6 | 25 |
| Pseudomonas aeruginosa isolate M | clinical isolate | PRJEB5438 | BUS44_RS30655 | 96.6 | 25 |
| Pseudomonas aeruginosa isolate N | clinical isolate | PRJEB5438 | BUS09_RS24945 | 96.6 | 25 |
| Pseudomonas aeruginosa isolate NA10 | clinical isolate | PRJEB5438 | BUS13_RS01680 | 96.6 | 25 |
| Pseudomonas aeruginosa isolate NA11 | clinical isolate | PRJEB5438 | BUS40_RS30095 | 96.6 | 25 |
| Pseudomonas aeruginosa isolate NA31 | clinical isolate | PRJEB5438 | BUS01_RS30265 | 96.6 | 25 |
| Pseudomonas aeruginosa isolate O | clinical isolate | PRJEB5438 | BUS43_RS30715 | 96.6 | 25 |
| Pseudomonas aeruginosa isolate P | clinical isolate | PRJEB5438 | BUS50_RS02420 | 96.6 | 25 |
| Pseudomonas aeruginosa isolate Q | clinical isolate | PRJEB5438 | BUS25_RS28875 | 96.6 | 25 |
| Pseudomonas aeruginosa isolate R | clinical isolate | PRJEB5438 | BUS27_RS30635 | 96.6 | 25 |
| Pseudomonas aeruginosa isolate S | clinical isolate | PRJEB5438 | BUS29_RS30140 | 96.6 | 25 |
| Pseudomonas aeruginosa isolate T | clinical isolate | PRJEB5438 | BUS38_RS26360 | 96.6 | 25 |
| Pseudomonas aeruginosa isolate U | clinical isolate | PRJEB5438 | BUS14_RS21610 | 96.6 | 25 |
| Pseudomonas aeruginosa isolate V | clinical isolate | PRJEB5438 | BUS03_RS29540 | 96.6 | 25 |
| Pseudomonas aeruginosa isolate X | clinical isolate | PRJEB5438 | BUS11_RS18745 | 96.6 | 25 |
| Pseudomonas aeruginosa isolate Y | clinical isolate | PRJEB5438 | BUS23_RS19250 | 96.6 | 25 |
| Pseudomonas aeruginosa isolate Z | clinical isolate | PRJEB5438 | BUS22_RS24010 | 96.6 | 25 |
| Pseudomonas aeruginosa XDR-PA / assembly BAL02-023 | clinical isolate | PRJEB31047 | E4Z17_RS25795 | 96.6 | 25 |
| Pseudomonas aeruginosa isolate BS2365 | environmental isolate | PRJEB32907 | F8N77_RS08815 | 96.6 | 25 |
| Pseudomonas aeruginosa isolate 124.1 | clinical isolate | PRJEB5438 | BUG59_RS05340 | 97.1 | 21 |
| Pseudomonas aeruginosa isolate 126.1 | clinical isolate | PRJEB5438 | BUG71_RS24625 | 97.1 | 21 |
| Pseudomonas aeruginosa isolate 127.1 | clinical isolate | PRJEB5438 | BUG07_RS23070 | 97.1 | 21 |

| | | | | | |
|---|---|---|---|---|---|
| Pseudomonas aeruginosa isolate 133_Pseudo_aeruginosa | clinical isolate | PRJEB5438 | BUG11_RS17600 | 97.1 | 21 |
| Pseudomonas aeruginosa isolate 330 | clinical isolate | PRJEB5438 | BUI84_RS16630 | 97.1 | 21 |
| Pseudomonas aeruginosa isolate 332 | clinical isolate | PRJEB5438 | BUJ94_RS02795 | 97.1 | 21 |
| Pseudomonas aeruginosa isolate 333 | clinical isolate | PRJEB5438 | BUI75_RS22910 | 97.1 | 21 |
| Pseudomonas aeruginosa isolate 334 | clinical isolate | PRJEB5438 | BUI52_RS15115 | 97.1 | 21 |
| Pseudomonas aeruginosa isolate 336 | clinical isolate | PRJEB5438 | BUI60_RS20370 | 97.1 | 21 |
| Pseudomonas aeruginosa isolate 337 | clinical isolate | PRJEB5438 | BUJ78_RS26160 | 97.1 | 21 |
| Pseudomonas aeruginosa isolate PcyII-10 | clinical isolate | PRJEB18612 | PERCYII10_RS19250 | 97.1 | 21 |
| Pseudomonas aeruginosa XDR-PA / assembly ARA01-045 | clinical isolate | PRJEB31047 | E4Y96_RS15870 | 97.1 | 21 |
| Pseudomonas aeruginosa XDR-PA / assembly CLM02-046 | clinical isolate | PRJEB31047 | E4Z83_RS17530 | 97.1 | 21 |
| Pseudomonas aeruginosa isolate RW130 | environmental isolate | PRJEB8749 | AQT41_RS08925 | 97.1 | 21 |
| Pseudomonas aeruginosa C2159M, isolate C2159M | clinical isolate | PRJNA232482 | Y905_RS23090 | 97.2 | 20 |
| Pseudomonas aeruginosa XDR-PA / assembly MAD04-014 | clinical isolate | PRJEB31047 | E4019_RS26030 | 97.2 | 20 |
| Pseudomonas sp HMSC067F09 | clinical isolate | PRJNA296326 | HMPREF2839_RS06810 | 97.2 | 20 |
| Pseudomonas sp. 2_1_26 | clinical isolate | PRJNA40037 | HMPREF1030_03139 | 97.2 | 20 |
| Pseudomonas denitrificans 461_PDEN | clinical isolate | PRJNA267549 | ADD34_RS04920 | 98.9 | 8 |
| Pseudomonas aeruginosa isolate 15.211Gc | environmental isolate | PRJEB8749 | AQT00_RS21635 | 98.9 | 8 |
| Pseudomonas aeruginosa isolate RW176 | environmental isolate | PRJEB8749 | AQR73_RS19670 | 98.9 | 8 |

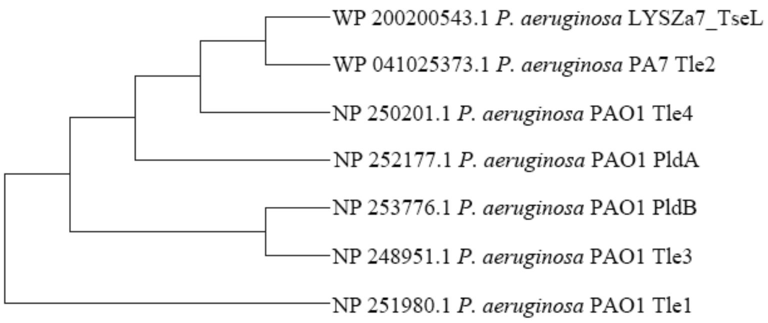


**Figure S1.** Phylogenetic analysis of LYSZa7_TseL with identified/putative Tle1-5 in *P. aeruginosa*.

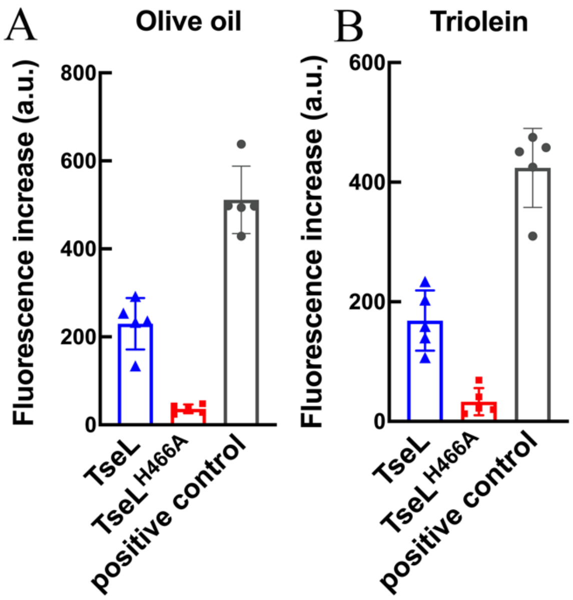


**Figure S2**. Lipase-activity of TseL$^{PA}$ and TseL$^{H466A}$ using (A) olive oil and (B) triolein as substrate (n=5). The enzyme activity using olive oil or triolein as substrates was monitored by using rhodamine B (0.001% w/v) as indicator, that fluorescence reading was determined with Ex/Em at 350 nm/580 nm.

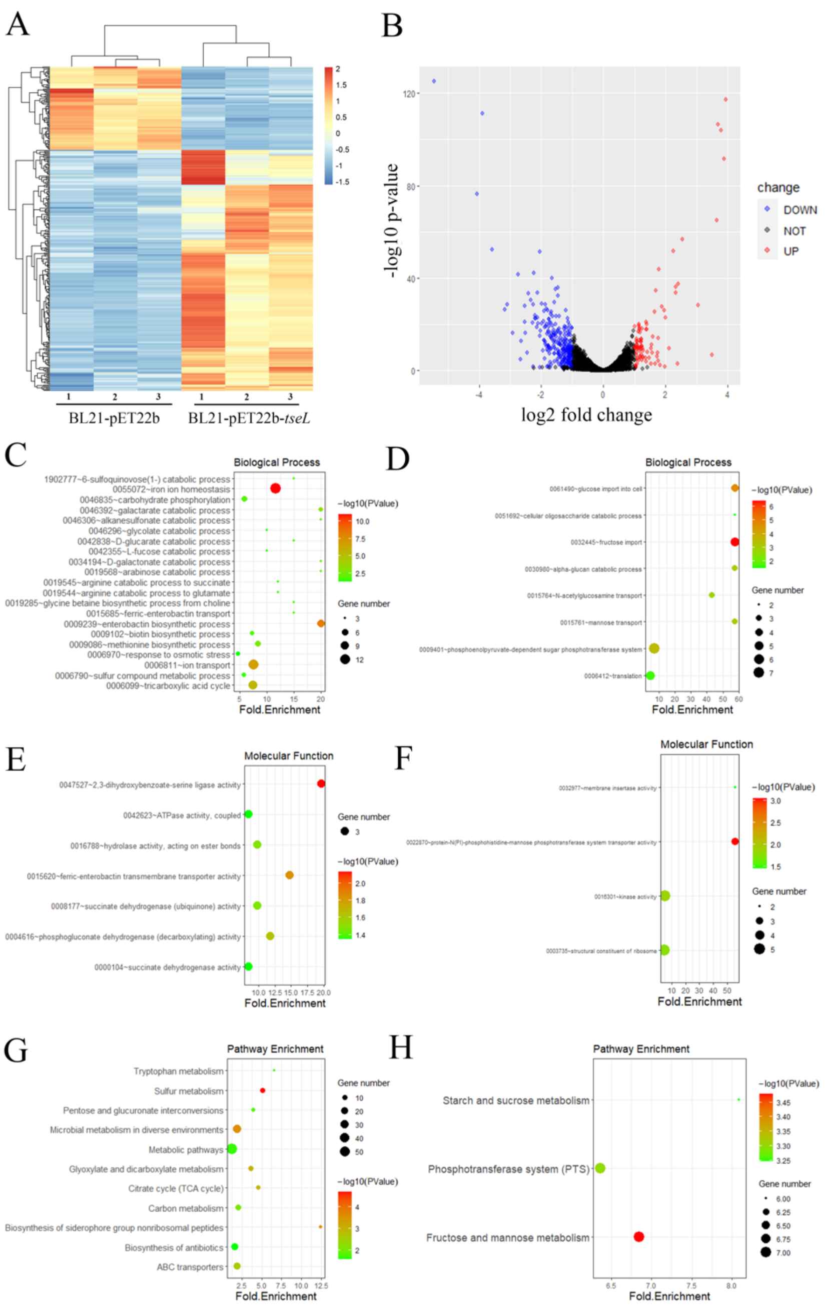


**FigureS3.** Transcriptomic profiling of *E. coli* expressing TseL$^{PA}$ was analyzed in comparison with *E. coli* harboring vector. **A.** Heatmap analysis of the dysregulated genes. **B.** Volcano plot of the significant genes. Enrichment analysis of up-regulated genes: (**C**) Biological process, (**D**) Metabolic pathways, and (**E**) cellular components. Enrichment analysis of down-regulated genes: (**F**) Biological process, (**G**) Molecular function and (**H**) Metabolic pathways

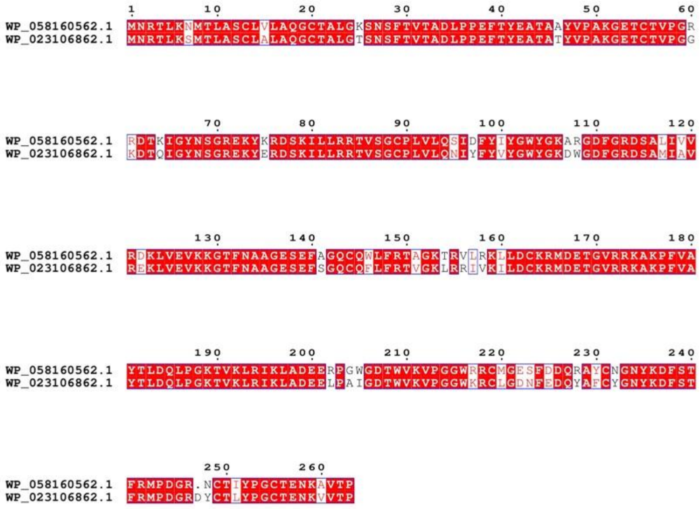

**Figure S4.** Protein sequence alignment of TsiP1 (WP_058160562.1) and TsiP2 (WP_023106862.1).

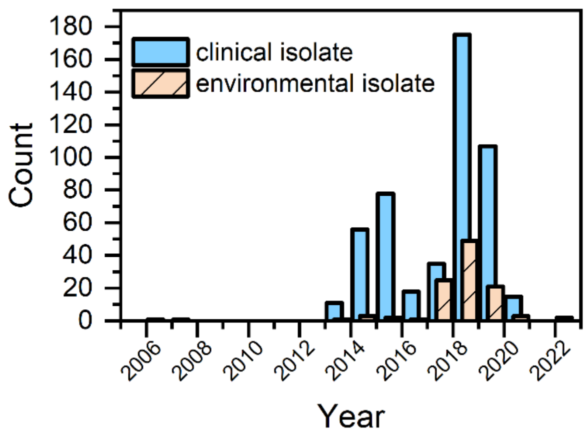


**Figure S5.** The year distribution of clinical and environmental *Pseudomonas* isolates which are able to encode TseL$^{PA}$ protein. In total, there are 683 isolates in the *Pseudomonas* Genome DB harboring TseL$^{PA}$, among which 573 are clinical isolates.

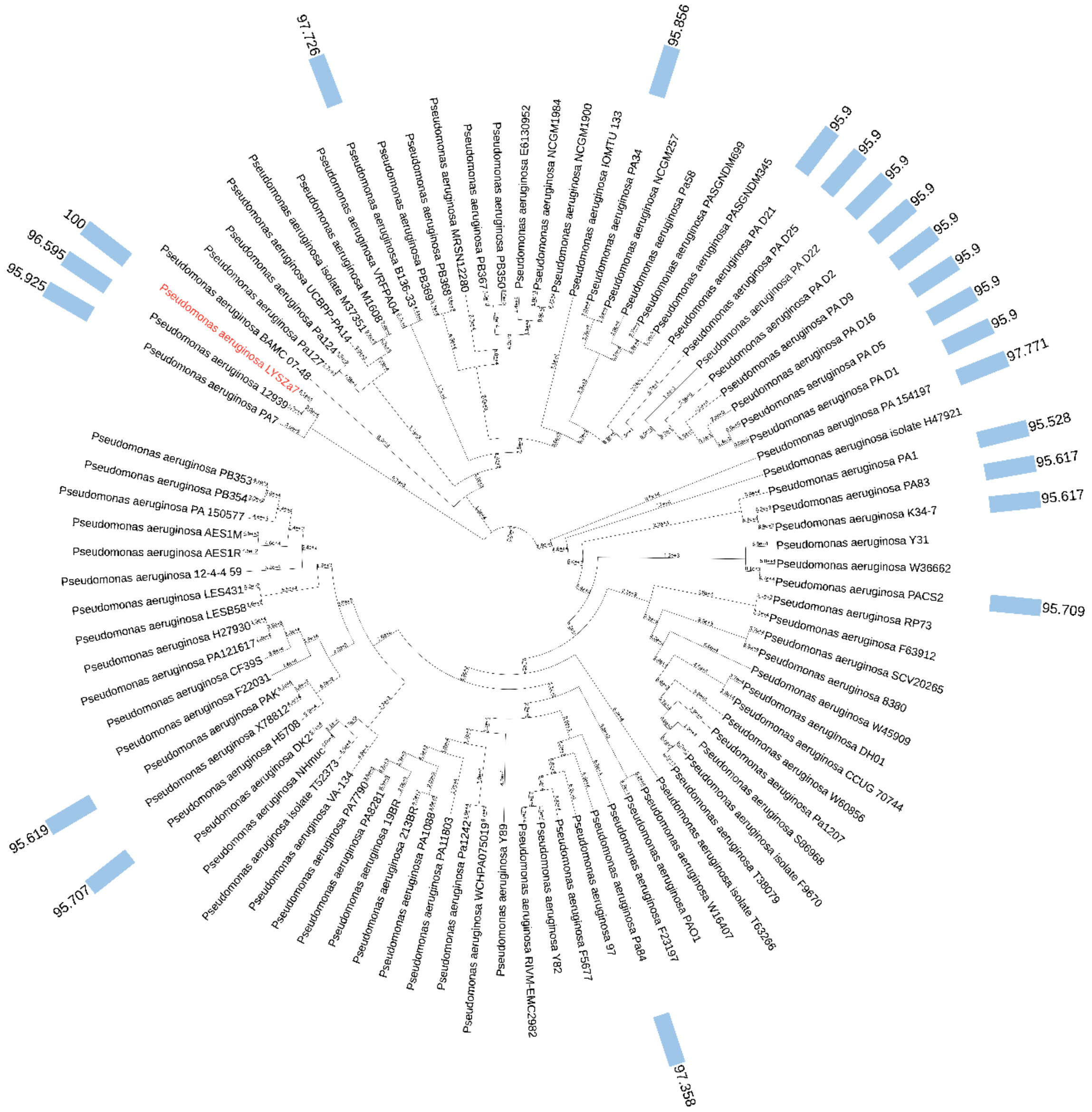


**Figure S6.** Presence of TseL$^{PA}$ in *P. aeruginosa* associated with human disease. There are 83 clinical isolates from *Pseudomonas* Genome DB by filtering out those with missing information in "host diseases". And 21 isolates (~25.3%) encode TseL$^{PA}$ protein (represent by the blue squares).
